# Supplementary material for: Intracellular signaling in proto-eukaryotes evolves to alleviate regulatory conflicts of endosymbiosis
Source: PLoS Comput Biol. 2024 Feb 9;20(2):e1011860. doi: 10.1371/journal.pcbi.1011860 (PMC10883579; doi:10.1371/journal.pcbi.1011860)
Supplement: S1 Appendix — (PDF) [file pcbi.1011860.s001.pdf]

S1 Appendix. Supplementary materials for Intracellular signalingin proto-eukaryotes evolves to alleviate regulatoryconflicts of endosymbiosis.

Samuel H. A. von der Dunk[1*], Paulien Hogeweg[1], Berend Snel[1]

[1]Department of Biology, Utrecht University, The Netherlands

February 5, 2024

Supplementary Results

Text A. Endosymbiosis between pre-adapted free-living cells leads to implicit control

All the experiments described in the main text were initiated with identical and primitive host and symbiont genomes. In the light of eukaryogenesis, it is interesting to compare these results with evolution experiments starting with pre-evolved and diverged lineages of "prokaryotes". To this end, we setup an evolution experiment with 12 different host–symbiont pairs, picking different prokaryotes that already adapted to poor nutrient conditions in an earlier study (1) (see Tables A and B). For every host–symbiont combination, three replicates were run which only differed in the random seed. We can use these triplicates to distinguish variation due to the host–symbiont pair from variation specific to a single run. To assess the impact of product leakage and gene transfer, we performed the whole experiment under different conditions: with leakage, transfer and signal peptide mutations (X1–12); with leakage only (L1–12); with transfer only (A1–12); and without leakage, transfer or signal peptide mutations (C1–12, see 2).

In the evolution experiments with pre-evolved hosts and symbionts, product leakage and gene transfer have a negative impact on final population size (Fig A). Across the 36 replicates evolved under leakage, transfer and signal peptide mutations, holobionts either went extinct ($N = 6$) or evolved implicit control ($N = 30$, identified by studying ancestral lineages viz. Fig L)—rather than any form of signaling—to coordinate host and symbiont

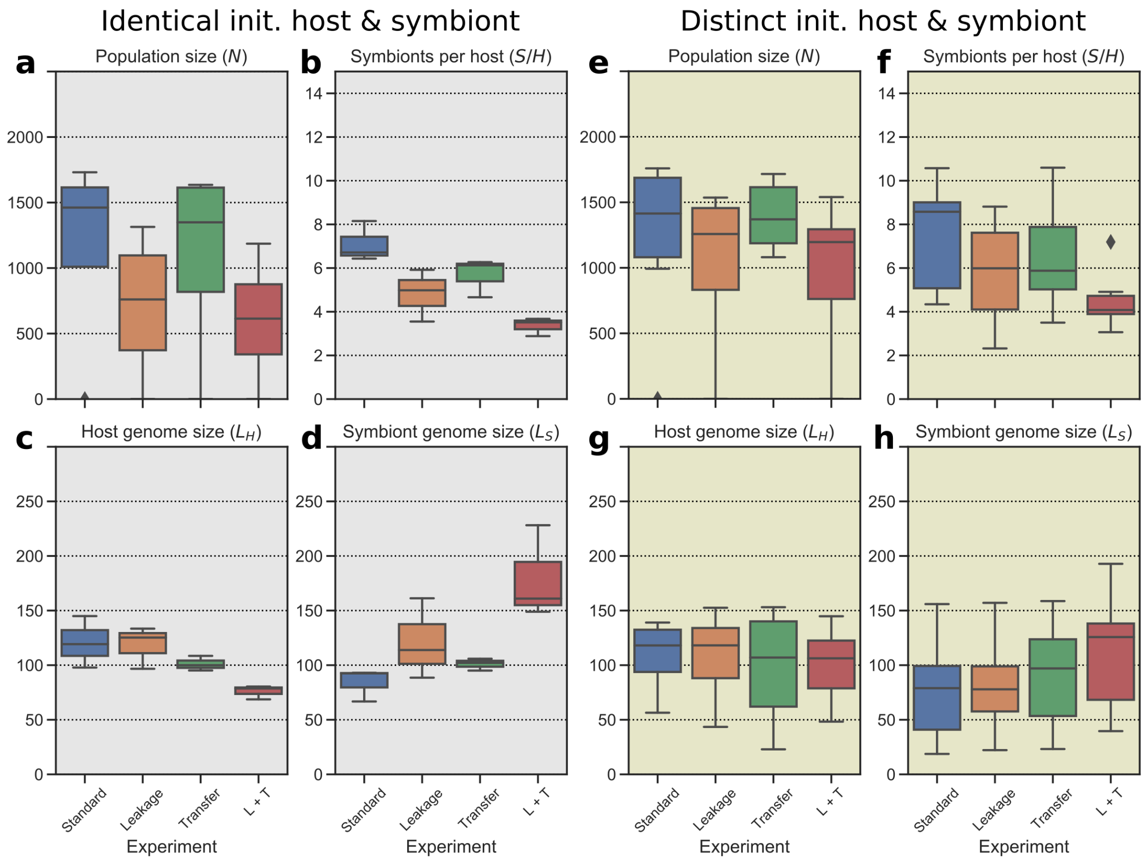

Figure A: Product leakage and gene transfer are deleterious for holobionts consisting of pre-evolved host–symbiont pairs, as seen in smaller final population sizes (a,e) and larger genomes (c,g and d,h) at $t = 2 \cdot 10^6$. These effects can be explained by the reduced symbiont numbers (b,f) which holobionts evolved to limit the effective leakage and transfer rates from symbiont to host. The impact of leakage is most negative on holobionts initialized with identical host and symbiont genomes (a–d) compared to holobionts initialized with distinct host and symbiont genomes (e–h). Standard refers to the experiment without leakage and transfer (see 2).

cell-cycles. Thus, signaling only emerges when relatively primitive hosts and symbionts (i.e. which are not adapted to poor nutrient conditions) are exposed to product leakage from the start.

Holobiont populations remain smaller in the presence of product leakage compared to the absence of product leakage, even after having evolved for $t = 2 \cdot 10^6$ timesteps (Fig Aa,e). The main adaptation to product leakage, and to a lesser extent to gene transfer, is to limit symbiont number (Fig Ab,f), reducing the effective rates of leakage and transfer. Symbiont numbers are limited by increasing the cell-cycle duration of the symbiont relative to the host (2), such that symbiont numbers are always diluted over multiple holobiont generations and are kept just above the bare minimum by holobiont-level selection. A slow symbiont cell cycle relative to that of the host relaxes selection for short symbiont genomes. Consequently, symbiont genomes expand more under the presence of leakage, and

even grow bigger than host genomes (Fig Ac,d,g,h). Interestingly, the small populations of symbionts with large genomes inside each holobiont are more sensitive to Muller's ratchet (2). In particular, the formation and modification of regulatory genes on the symbiont genome is a hazard because these may subsequently leak or transfer to the host genome.

The impact of leakage and transfer is largest when evolution is started with identical host and symbiont genomes (Fig A), i.e. similar to the situation in the experiments described in the main text. When host and symbiont genomes start out identical, genes retain some of their ancestral overlap, resulting in the strongest molecular interference. The average Hamming distance between the binding sequences of the g5 copies in host and symbiont after $t = 2 \cdot 10^6$ timesteps is $\bar{d} = 1.78$ ($N = 9$) for the replicates starting with identical host and symbiont and $\bar{d} = 6.29$ ($N = 21$) for the replicates starting with different host and symbiont. This means that in replicates starting with identical host and symbiont, binding sites for g5 in the symbiont remain very sensitive to the host version of g5 if it is leaked to the symbiont and vice versa. The interference remains in particular for gene g5 because it targets many binding sites which, as we previously showed, constrains the evolution of its binding sequence (see "Gene family analysis" in Appendix 1 of 1), in line with the evolutionary behavior of real transcription factors (3). Apparently, hosts and symbionts are unable to modify their gene regulatory network and to avoid the effects of leakage at the level of gene regulation.

Text B. Symbiont control strategies

To understand how different control mechanism achieve stabilization of symbiont numbers, we tracked individual cells growing at intermediate nutrient conditions ($n_{\text{influx}} = 30$), either through their normal division process or by enforcing symmetric distribution of symbionts. By analogy to the literature on cell size control (e.g. 4; 5; 6), wherein cell behavior can be analyzed by correlating the cell volumes between two consecutive cell cycles, we here compare the symbiont number right after division between two consecutive cell cycles (Fig B). Using this approach, the four different symbiont control strategies could be grouped into three different phenomenological control behaviors as mentioned in the main text.

Host control ensures replication of all symbionts during the holobiont cell cycle, suggesting that deviations in symbiont numbers are propagated to subsequent generations. In line with this description, there is a high correlation between symbiont numbers of consecutive generations (Fig Ba). Furthermore, the clonal growth experiments (i.e. Fig 4 in main text) show that holobiont deaths are much more frequent with host control than in any

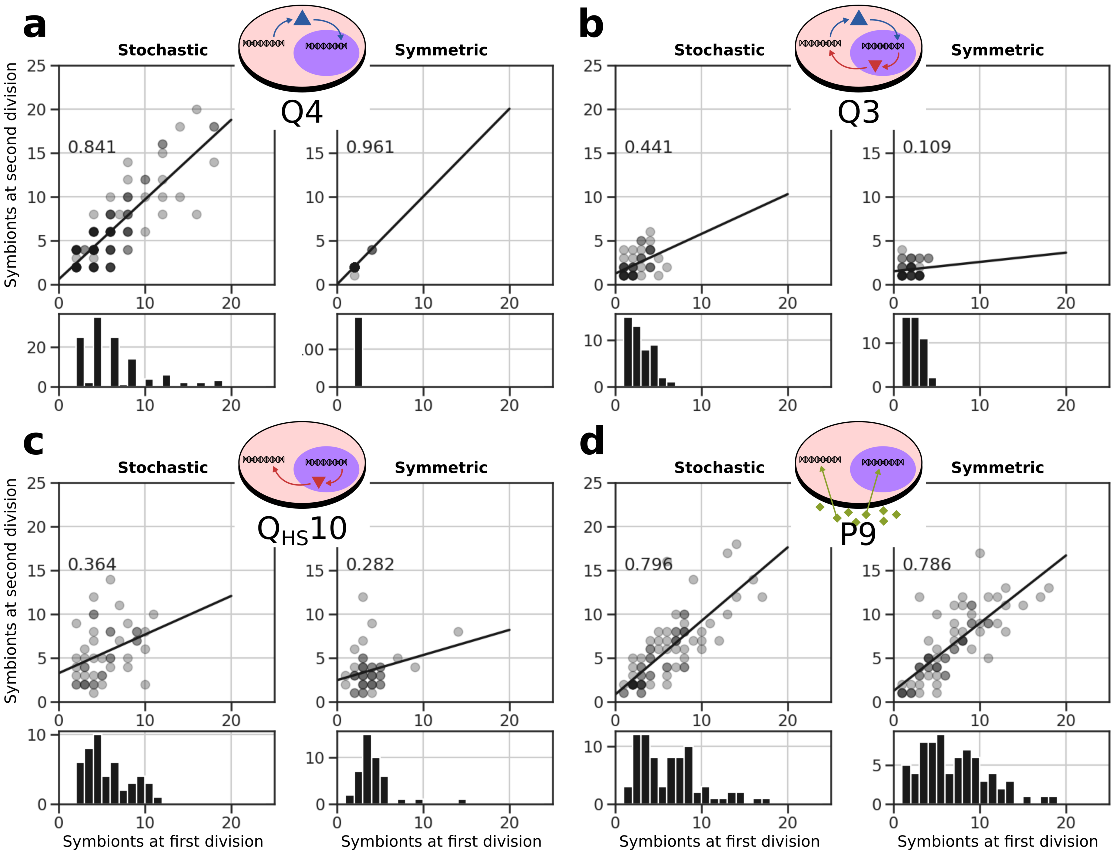

Figure B: Phenomenological control strategies identified by correlations in symbiont numbers between consecutive cell cycles. As in Fig 4 in main text, the most recent common ancestor of each replicate was analyzed at intermediate nutrient conditions ($n_{\text{influx}} = 30$). Removing the stochasticity at cell division by forcing symmetric division shows the control strategy more clearly (right versus left panels). In the case of P9, the correlation stays well below 1, indicating that holobionts approach the equilibrium symbiont number over multiple generations. See also Fig C.

of the other control mechanisms (effective rate of 0.00843 in Q4 with host control versus 0.00361 for Q3 with bi-directional control, 0.00282 for $Q_{HS}10$ with symbiont control and 0.00256 for P9 with implicit control), indicating that holobiont-level selection is important for stabilizing symbiont numbers in the population. At the same time, births are also more frequent with host control (effective rate of 0.0225 in Q4 versus 0.0094 for Q3, 0.0078 for $Q_{HS}10$ and 0.0114 for P9), indicating that holobiont-level selection acts rapidly and without detriment to the population.

In contrast, bi-directional control and symbiont control establish division checkpoints that stall the cell cycle until sufficiently many symbionts are present. In line with this mechanism, the correlation between symbiont numbers in consecutive generations is small (Fig Bb,c). Strikingly, the removal of stochasticity at cell division, i.e. by enforcing symmetric symbiont distribution, makes the control behaviors of host control on one hand,

and of bi-directional control and symbiont control on the other hand, even clearer.

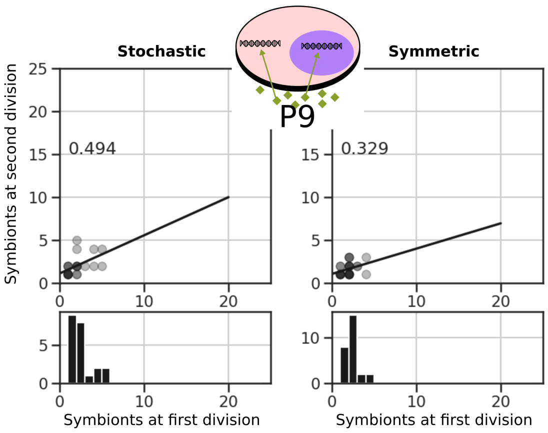

Figure C: Phenomenological control strategy of P9 becomes more apparent at poor nutrient conditions ($n_{\text{influx}} = 5$) where due to low symbiont numbers, the impact of stochasticity at cell division is more prominent (cf. Fig B).

Finally, implicit control constitutes a weak sizer that takes several generations to correct deviations in symbiont number. For this reason, we do not find such a clear sizer signal as with bi-directional control or symbiont control (Fig Bd). In particular, the impact of stochasticity in symbiont number at division is relatively small for holobionts with high symbiont number, such that the correlation only decreases very slightly when symmetric distribution is enforced. Still, when we focus on poor nutrient conditions where holobionts have few symbionts such that symmetric distribution has a large impact, we retrieve the sizer signature that was also found for bi-directional control and symbiont control: without stochasticity at cell division, the correlation between symbiont numbers in consecutive cell cycles is decreased (Fig C).

Text C. Symbiont control by two separate checkpoints for host division

In both Q3 and $Q_{\text{HS}}10$ where bi-directional and symbiont control strategies evolved, symbionts signal to the host when their numbers are high enough inciting the host to safely divide. A key difference with bi-directional control in Q3 is that the interaction between symbiont g2 and host g7 in $Q_{\text{HS}}10$ is not dosage-sensitive and does not measure the replication status of the host (Figs 4 and U). The host has evolved a second mechanism to infer the replication status of its own genome. A gene near the origin, g13, performs the same regulatory tasks as g7, and thus prevents host division even if g7 is deactivated under the influence of high symbiont number. Instead, g13 needs to be inhibited by g5, which is located right at the terminus of the host genome. The g5–g13 interaction depends on low-affinity binding, so inhibition of g13 only becomes likely when the entire host genome

is replicated (giving two copies of g5). Thus, where bi-directional control in Q3 integrates host status and symbiont levels into a single cell-cycle checkpoint, symbiont control in $Q_{HS}10$ rests on two independent checkpoints for holobiont division.

Supplementary Tables and Figures

Table A: Important characteristics of pre-evolved free-living prokaryotes, i.e. cell-cycle efficiency (see Fig 6 in main text) and generalist capacity (plasticity measured as the log-difference between cell-cycle duration at $n = 0.1$ and $n = 100$).

Strain	Efficiency (e)	Plasticity (σ)
R8	0.476	0.965
R9	0.402	0.866
R2	0.307	-0.355
R3	0.160	0.497

Table B: Host–symbiont pairs used to initialise evolution experiments (see Table A for characteristics of prokaryotes). C1–4 start with identical host and symbiont; C5–6 have different host and symbiont but with very similar phenotypic behavior, i.e. R8 and R9 are both efficient generalists; C7–12 are asymmetric.

Holobiont	Host	Symbiont
C1	R8	R8
C2	R9	R9
C3	R2	R2
C4	R3	R3
C5	R8	R9
C6	R9	R8
C7	R8	R2
C8	R2	R8
C9	R3	R2
C10	R2	R3
C11	R8	R3
C12	R3	R8

Table C: Genetic sequences of the five core gene types in host and symbiont of the most recent common ancestor of Q4 which lived at $t = 8.9 \cdot 10^6$.

Gene	Host	Symbiont	Hamming distance (d)
g1	10001101110111100010	11111100101110110101	11
g2	11100110101111000100	01001000010100110101	13
g3	01000111111110010010	00111001010010010101	12
g4	01100010001100010000	01010100100100011010	8
g5	10000110111010001011	01000111111110010100	9

References

[1] von der Dunk SH, Snel B, Hogeweg P. Evolution of Complex Regulation for Cell-Cycle Control. Genome Biology and Evolution. 2022;14(5):evac056.

[2] von der Dunk SH, Hogeweg P, Snel B. Obligate endosymbiosis enables genome expansion during eukaryogenesis. Communications Biology. 2023;6(1):777.

[3] Chesmore KN, Bartlett J, Cheng C, Williams SM. Complex patterns of association between pleiotropy and transcription factor evolution. Genome Biology and Evolution. 2016;8(10):3159–3170.

[4] Sauls JT, Li D, Jun S. Adder and a coarse-grained approach to cell size homeostasis in bacteria. Current Opinion in Cell Biology. 2016;38:38–44.

[5] Witz G, van Nimwegen E, Julou T. Initiation of chromosome replication controls both division and replication cycles in E. coli through a double-adder mechanism. Elife. 2019;8:e48063.

[6] Proulx-Giraldeau F, Skotheim JM, François P. Evolution of cell size control is canalized towards adders or sizers by cell cycle structure and selective pressures. Elife. 2022;11:e79919.

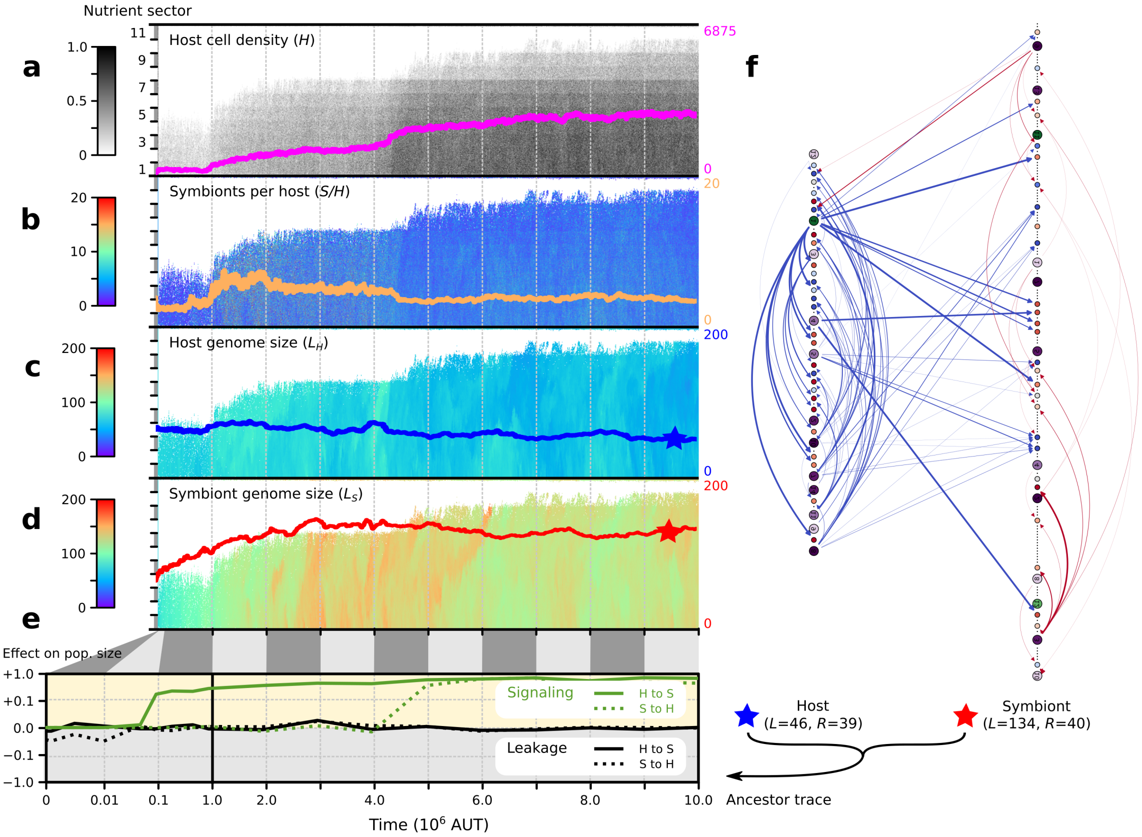

Figure D: Evolutionary dynamics of replicate Q3 where holobionts evolved bi-directional control (cf. Fig 2 in main text). Here, the symbiont evolves a substantially larger genome than the host. Yet, the sizes of their regulatory repertoires are very similar and the host controls more symbiont genes than vice versa. As in Q4, holobionts quickly evolve to be insensitive to leakage (bottom panel) and host-to-symbiont signaling also evolves rapidly. Only from $t = 4 \cdot 10^6$, symbiont-to-host signaling evolves yielding bi-directional control.

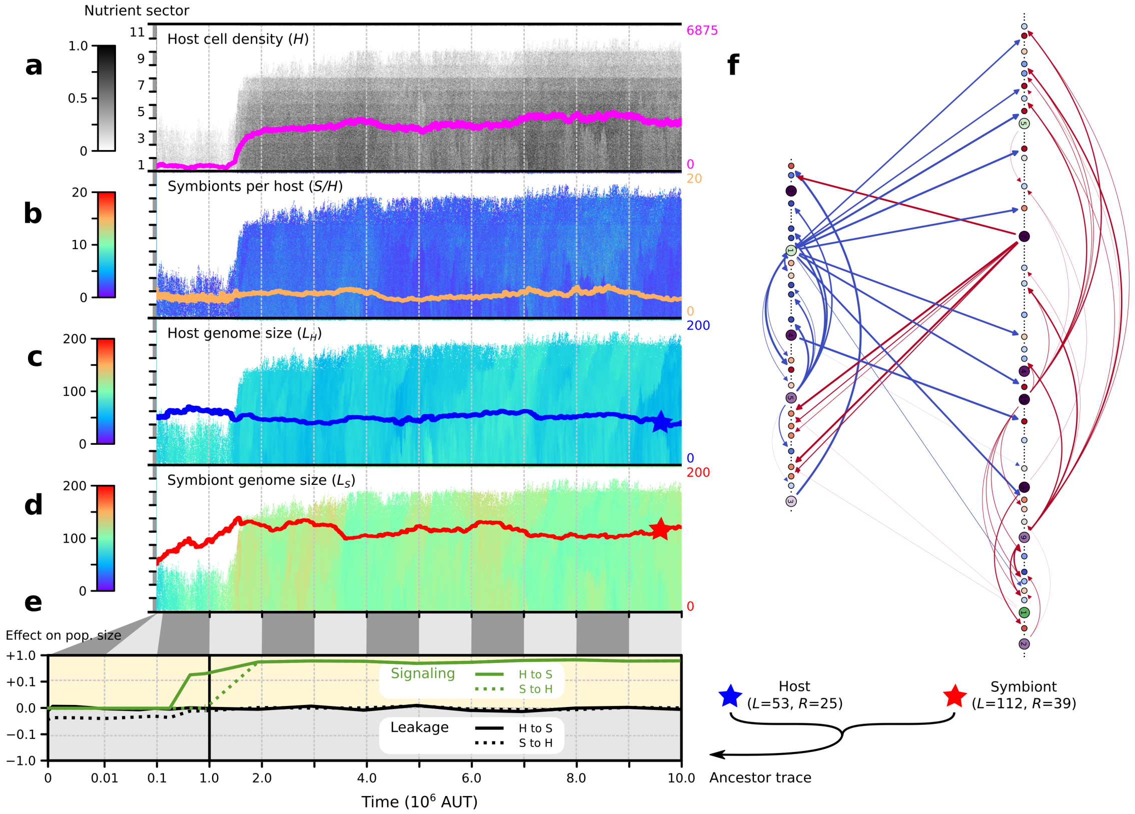

Figure E: Evolutionary dynamics of Q5, in which bi-directional control evolved.

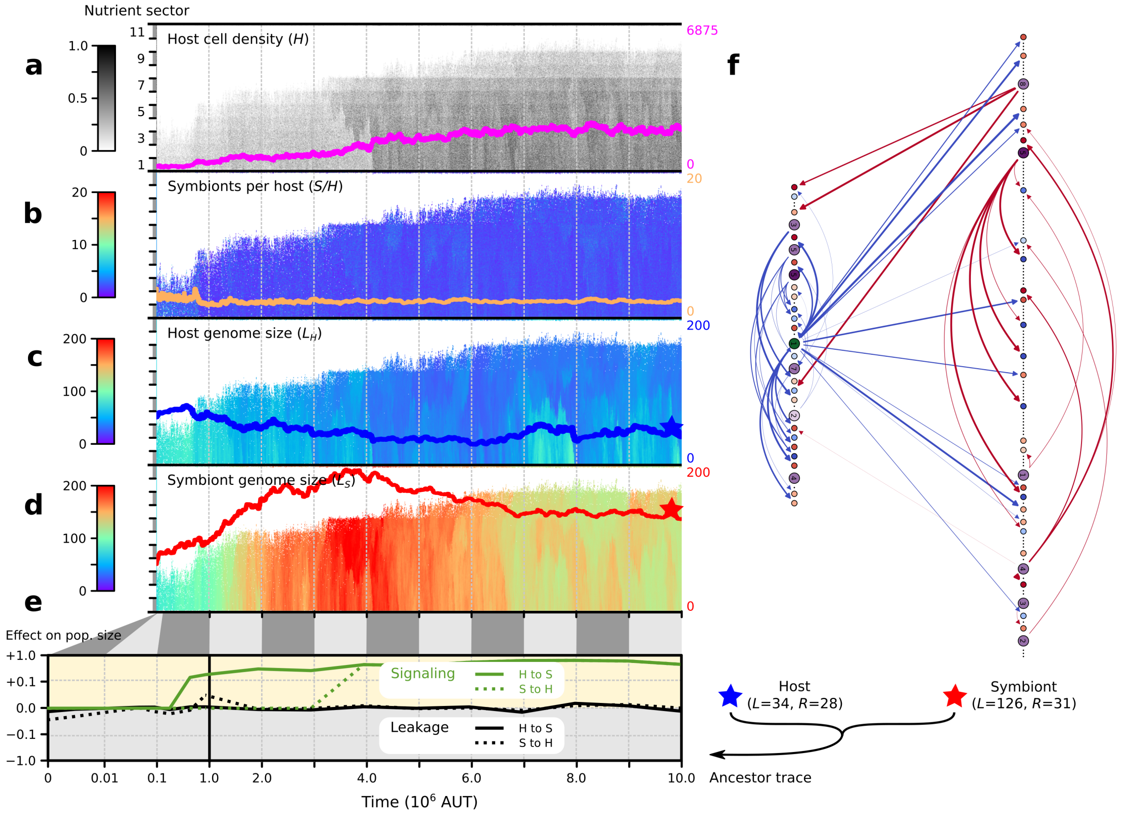

Figure F: Evolutionary dynamics of Q7, in which bi-directional control evolved.

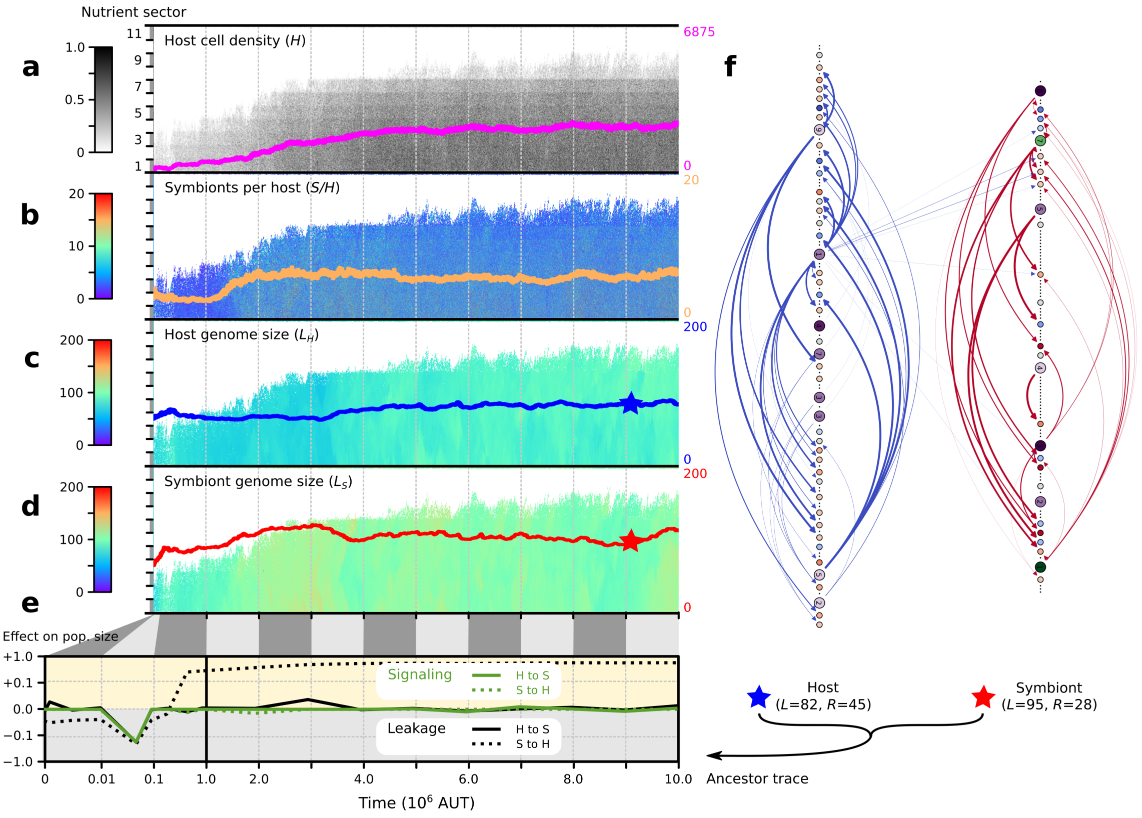

Figure G: Evolutionary dynamics of Q8, in which symbiont control evolved.

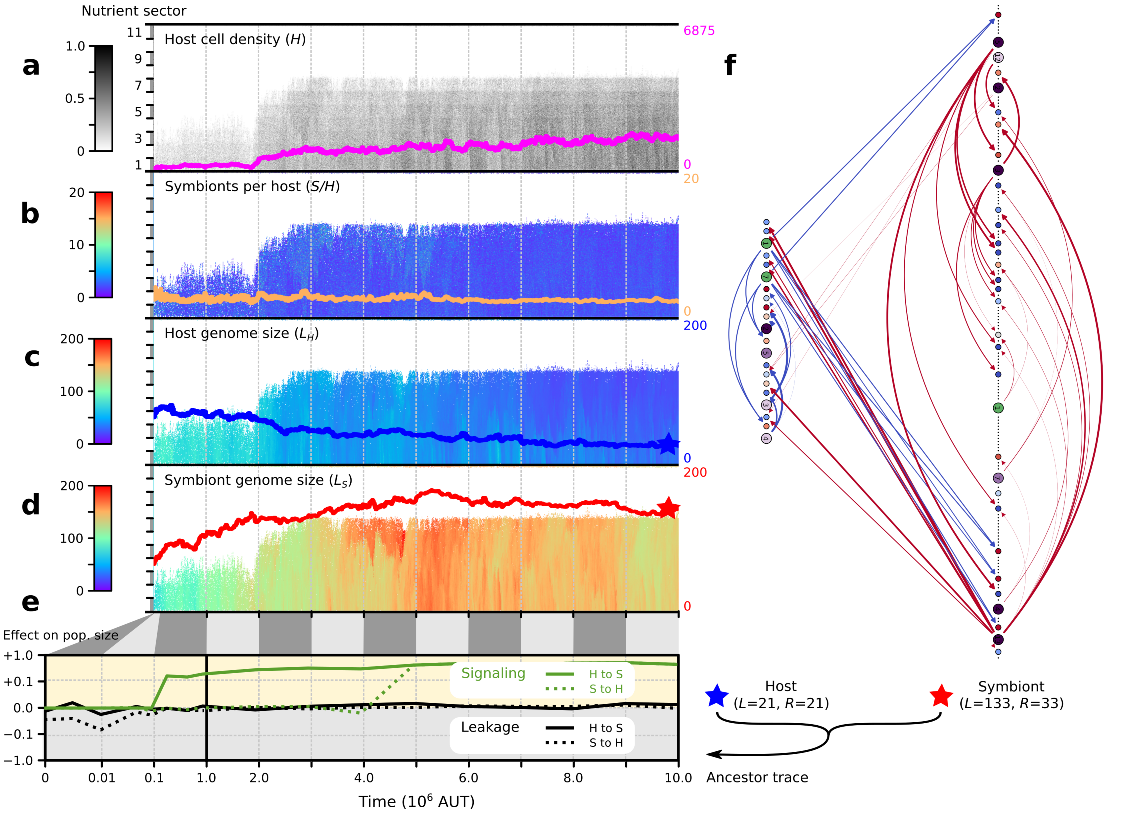

Figure H: Evolutionary dynamics of Q9, in which bi-directional control evolved.

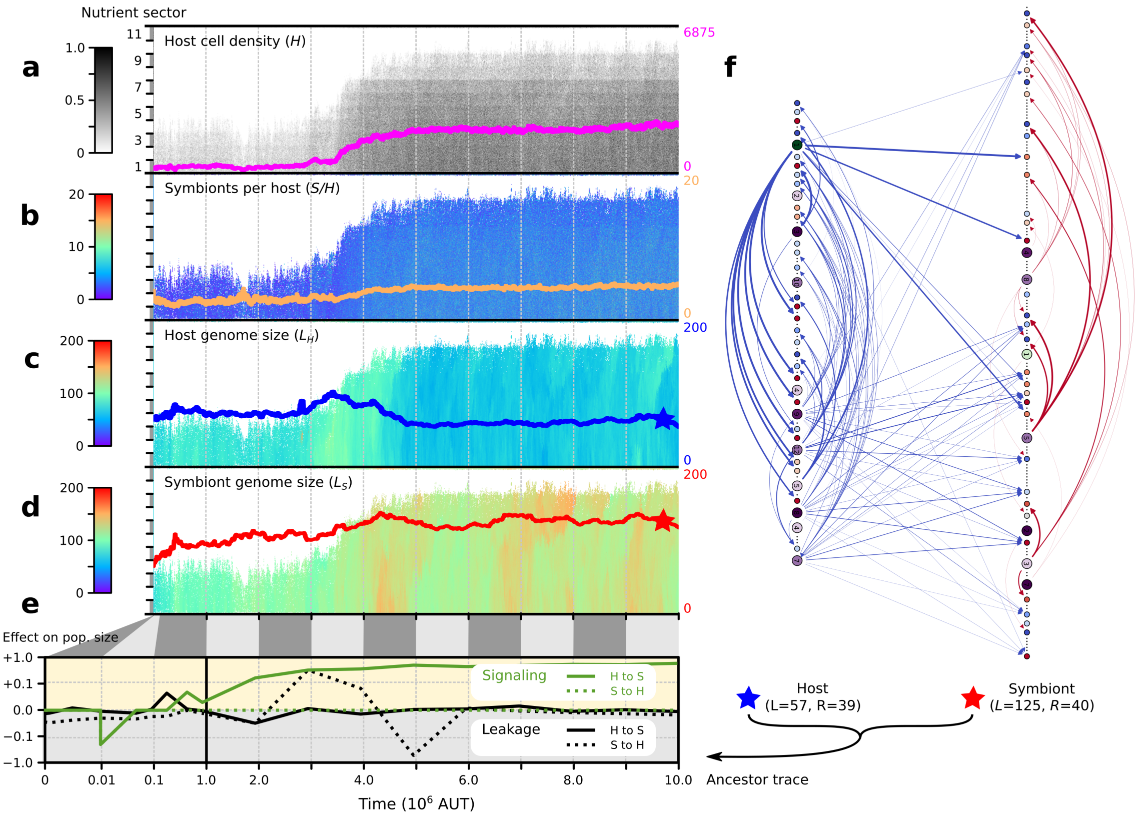

Figure I: Evolutionary dynamics of Q10, in which host control evolved.

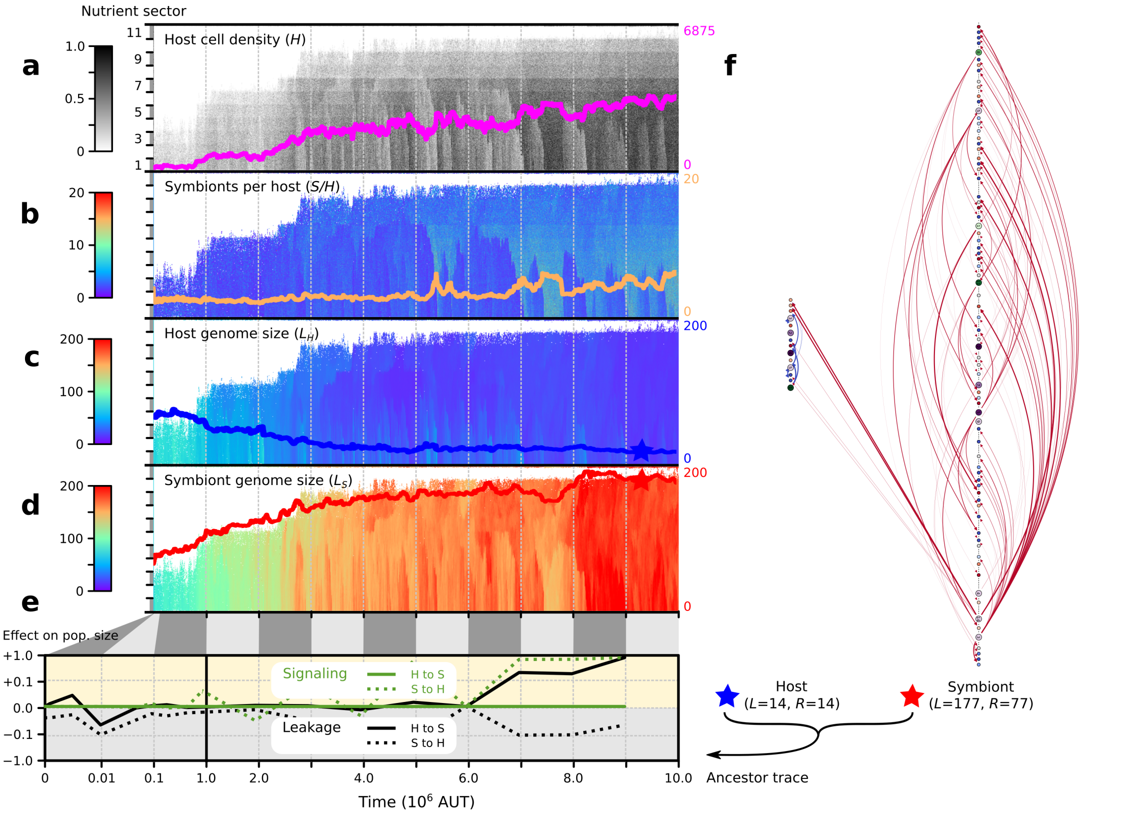

Figure J: Evolutionary dynamics in a replicate where holobionts evolved bi-directional control using host-to-symbiont leakage ($Q_{HS}2$).

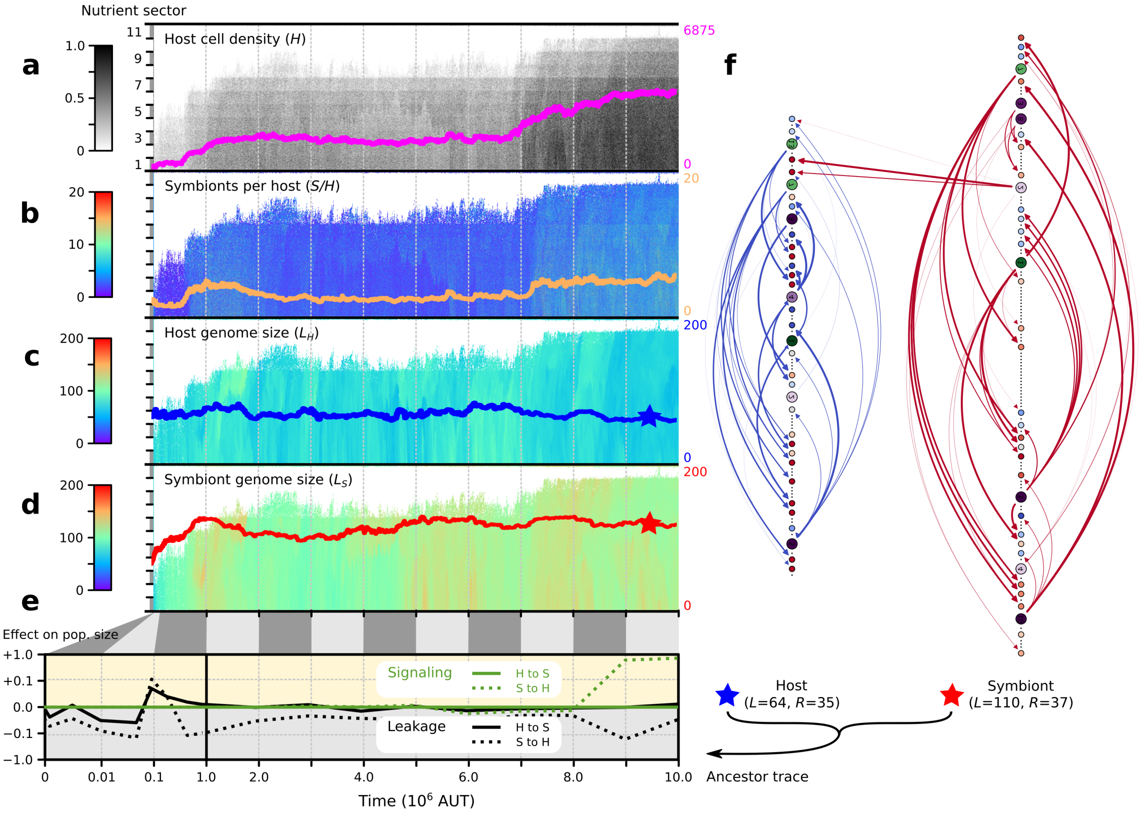

Figure K: Evolutionary dynamics in a replicate where holobionts evolved symbiont control ($Q_{HS}10$; cf. Fig 2 in main text). As in Q3, the symbiont evolves a larger genome than the host and the sizes of their regulatory repertoires are comparable. Symbiont-to-host signaling evolves remarkably late, coinciding with substantial population expansion. As before, holobionts quickly evolve to become insensitive to host-to-symbiont leakage (bottom panel). In contrast, holobionts were not exposed to symbiont-to-host leakage, so they remain sensitive to the introduction of this type of leakage.

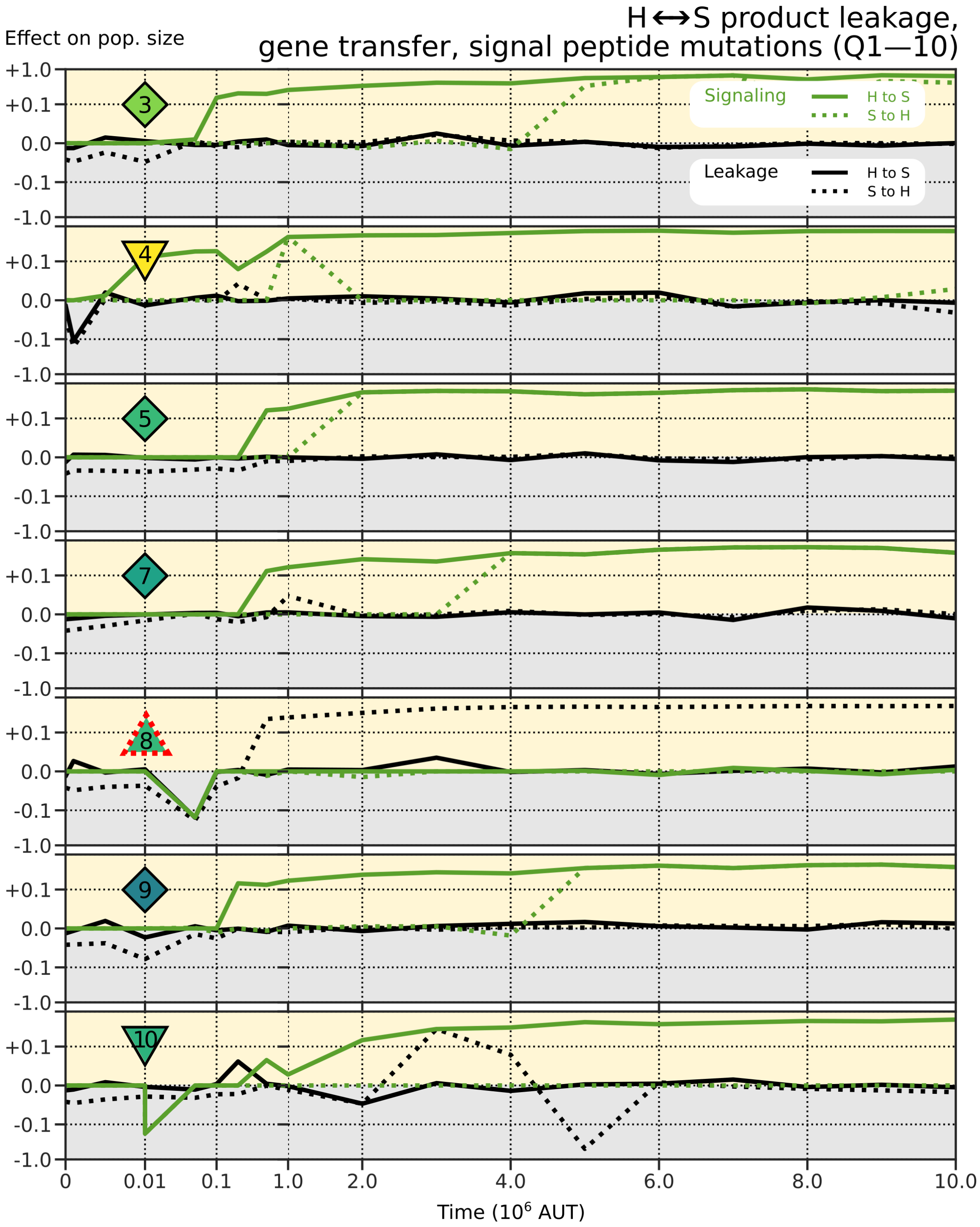

Figure L: Impact of leakage and signaling along ancestral lineages during evolution with product leakage, gene transfer and signal peptide mutations (Q1–10). Host-to-symbiont signaling always appears early in evolution or does not appear at all. In bi-directional control (Q3, Q5, Q7, and Q9), symbiont-to-host signaling evolves substantially later. In all replicates except Q8, holobionts rapidly become insensitive to leakage. Symbols of replicates correspond to those in Fig 3 in main text: Triangles indicate the direction of control (down for host control, up for symbiont control, diamond for bi-directional control), and red dotted lines indicate communication through leakage (down for host-to-symbiont direction, up for symbiont-to-host direction). Replicates were continued beyond $t = 10^7$, so we could determine the ancestral lineage up to $t = 10^7$.

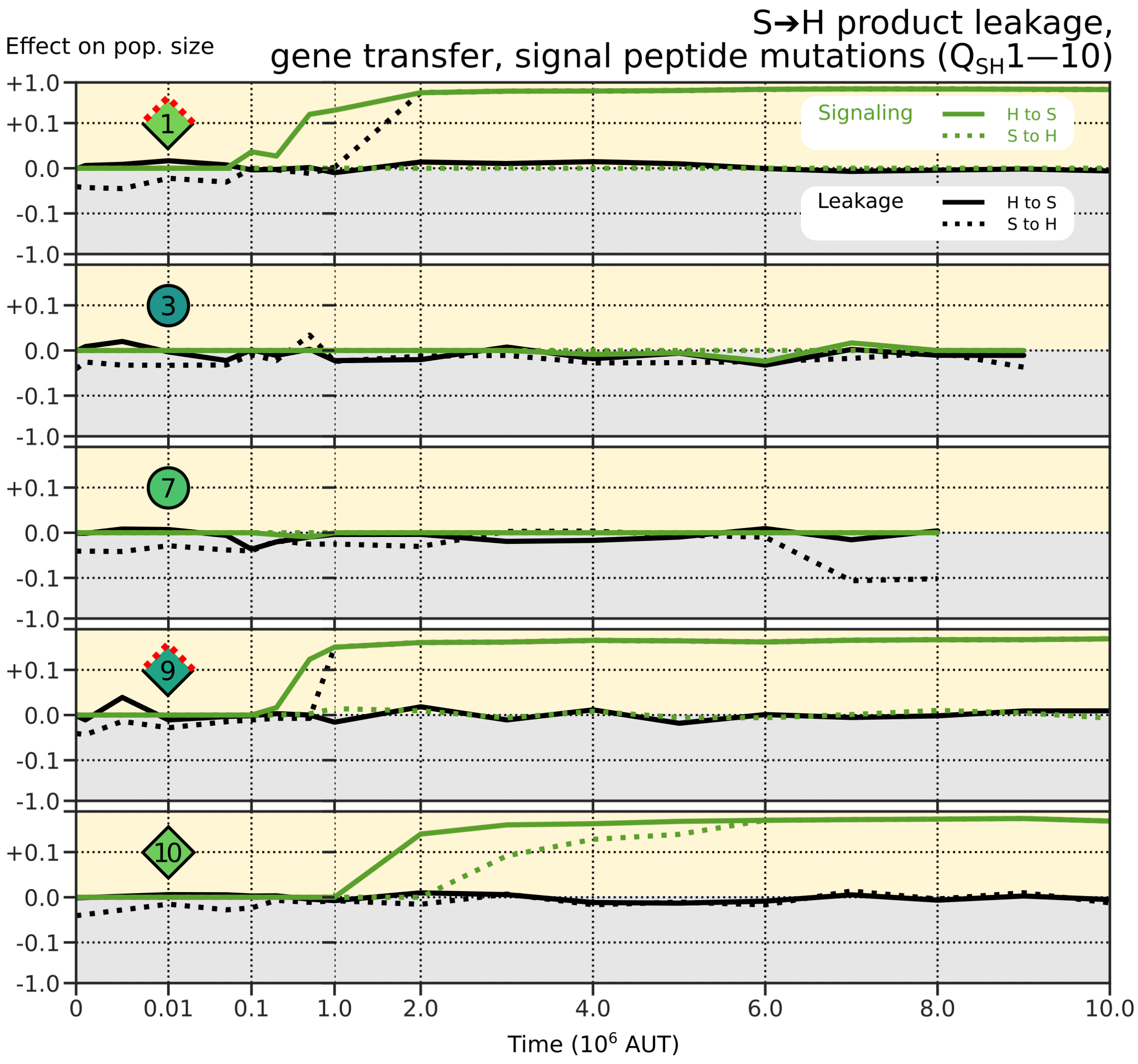

Figure M: Impact of leakage and targeting along ancestral lineages during evolution with symbiont-to-host product leakage, gene transfer and signal peptide mutations (Q_{SH}1–10). As in Fig L, host-to-symbiont communication arises before symbiont-to-host communication. Two replicates (Q3 and Q7) do not evolve any functional communication. Symbols of replicates correspond to those in Fig 3 in main text. Holobionts were not exposed to host-to-symbiont leakage, and leakage in this direction has little effect on evolved holobionts. Several replicates were continued beyond $t = 10^7$, so we could determine the ancestral lineage up to $t = 10^7$.

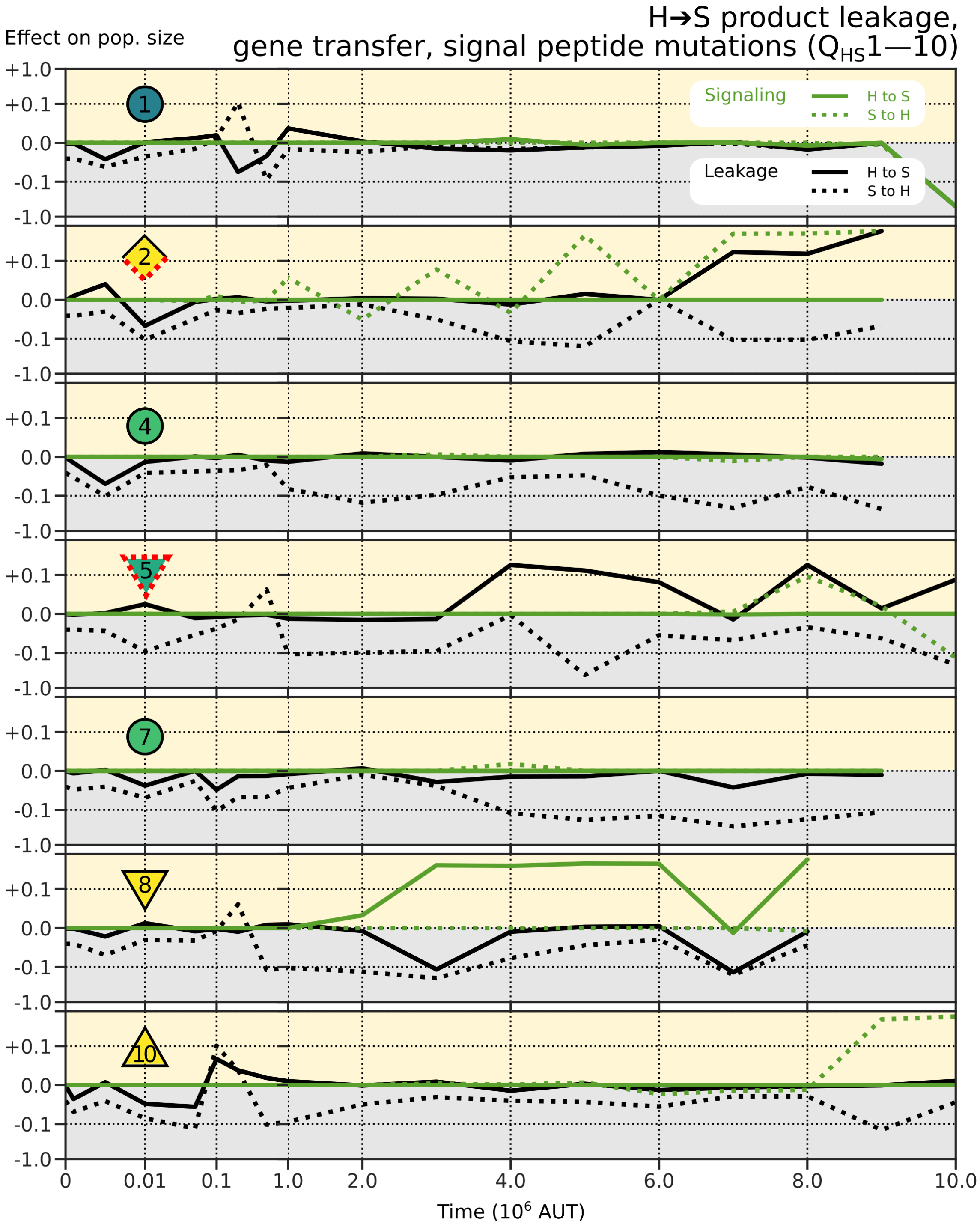

Figure N: Impact of leakage and targeting along ancestral lineages during evolution with host-to-symbiont product leakage, gene transfer and signal peptide mutations (Q_{HS}1–10). Unlike in replicates Q1–10 (Fig L), holobionts do not evolve under symbiont-to-host leakage and thus do not become insensitive to it. Symbols of replicates correspond to those in Fig 3 in main text. Several replicates were continued beyond $t = 10^7$, so we could determine the ancestral lineage up to $t = 10^7$.

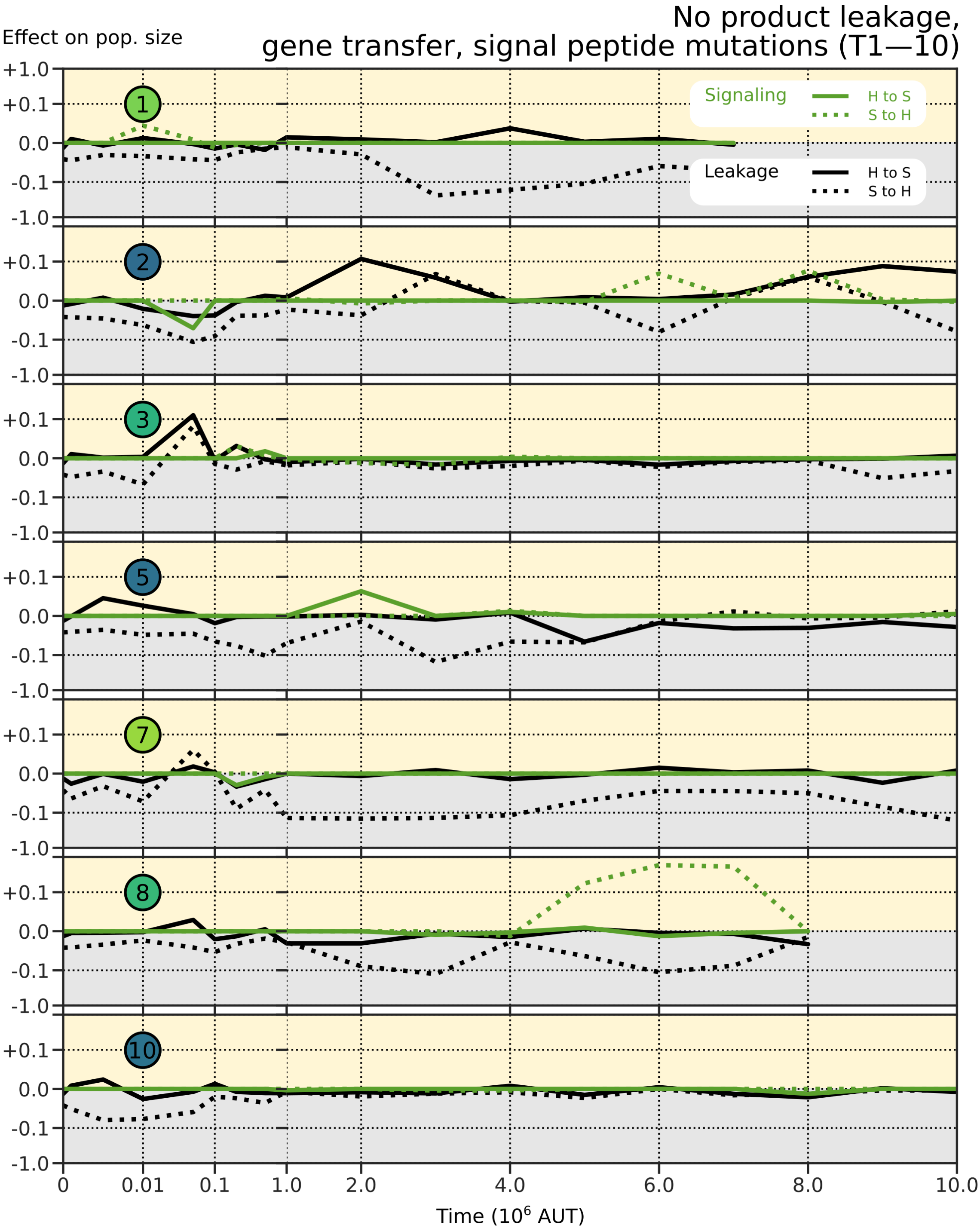

Figure O: Impact of leakage and targeting along ancestral lineages during evolution with gene transfer and signal peptide mutations but without leakage (T1–10). Functional signaling is only observed transiently in T8. In addition, holobionts do not evolve under leakage, and thus do not become insensitive to it. Still, holobionts might turn out to evolve a transient positive effect of leakage on growth (e.g. early on in T3). Symbols of replicates correspond to those in Fig 3 in main text. Several replicates were continued beyond $t = 10^7$, so we could determine the ancestral lineage up to $t = 10^7$.

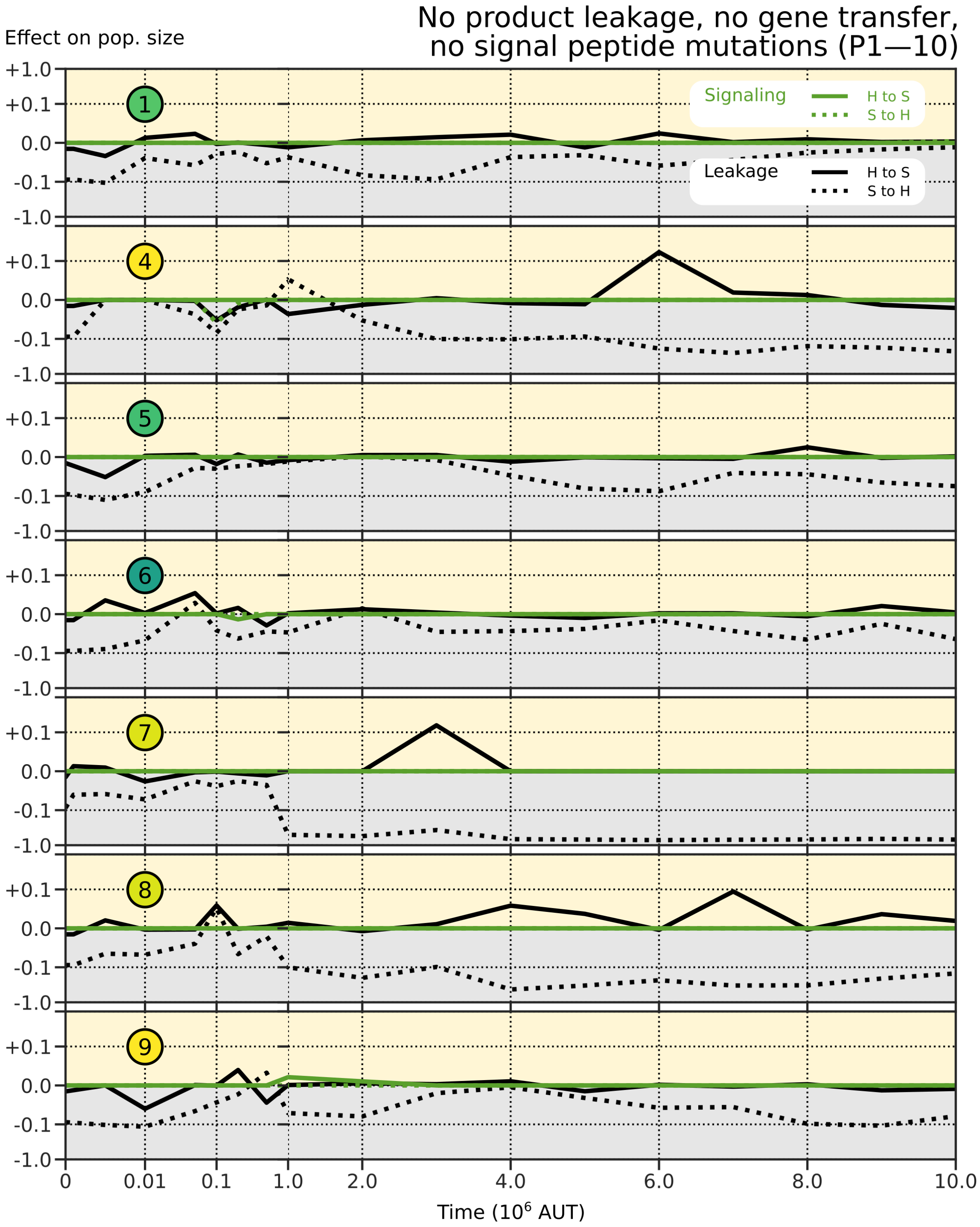

Figure P: Impact of leakage and targeting along ancestral lineages during evolution without any interference and without the possibility for communication between host and symbiont (P1–10). Symbols of replicates correspond to those in Fig 3 in main text. Holobionts were not exposed to leakage, and in many cases remain particularly sensitive to symbiont-to-host leakage. P7 evolves to be especially sensitive to symbiont-to-host leakage, fitting with the large population reduction observed in Fig 5 in main text. Conversely, host-to-symbiont leakage is relatively harmless, and even transiently beneficial in P4, P7 and P8. Several replicates were continued beyond $t = 10^7$, so we could determine the ancestral lineage up to $t = 10^7$.

	Q4	P9	P4	P8	P7	P1	Q10	P5	Q5	Q7	P6	Q3	Q9	Q8
**Q4**		0	0	9.8	12.2	14.71	28.57	27.03	28.57	30.3	43.48	23.26	62.5	47.62
**P9**	0		0	8.06	9.9	10.75	17.86	20.83	25.64	25	27.78	16.67	41.67	35.71
**P4**	0	0		6.8	6.76	12.05	15.87	18.52	21.28	21.28	34.48	11.9	43.48	37.04
**P8**	-9.8	-8.06	-6.8		0	5.95	11.24	14.71	18.18	20.41	24.39	8.4	34.48	29.41
**P7**	-12.2	-9.9	-6.76	0		7.09	11.49	10	15.15	12.05	19.23	8.4	37.04	35.71
**P1**	-14.71	-10.75	-12.05	-5.95	-7.09		5.13	6.06	7.94	5.92	16.13	38.46	28.57	25.64
**Q10**	-28.57	-17.86	-15.87	-11.24	-11.49	-5.13		1.28	3.37	6.29	9.09	0	22.22	25
**P5**	-27.03	-20.83	-18.52	-14.71	-10	-6.06	-1.28		0	2.08	2.83	0	15.38	21.74
**Q5**	-28.57	-25.64	-21.28	-18.18	-15.15	-7.94	-3.37	0		0	7.75	0	19.61	18.18
**Q7**	-30.3	-25	-21.28	-20.41	-12.05	-5.92	-6.29	-2.08	0		1.6	0	13.7	21.28
**P6**	-43.48	-27.78	-34.48	-24.39	-19.23	-16.13	-9.09	-2.83	-7.75	-1.6		0	7.81	25
**Q3**	-23.26	-16.67	-11.9	-8.4	-8.4	-38.46	0	0	0	0	0		1.58	16.95
**Q9**	-62.5	-41.67	-43.48	-34.48	-37.04	-28.57	-22.22	-15.38	-19.61	-13.7	-7.81	-1.58		15.87
**Q8**	-47.62	-35.71	-37.04	-29.41	-35.71	-25.64	-25	-21.74	-18.18	-21.28	-25	-16.95	-15.87	

Figure Q: Competition experiments between the 14 adapted populations of Q1–10 and P1–10 without leakage (cf. Fig R). Two populations are inoculated side-by-side (one at the top, one at the bottom) of an enlarged grid which fits both populations entirely (50×275). Each competition experiment lasts for 10^5 timesteps and includes mutations. The time at which one population has expelled the other from the grid is recorded and a draw is recorded if both populations are still present on the grid at the end. All values in the table denote competitive success of the population in the row relative to the population in the column ($s = \frac{10^5}{t_{\text{win}}}$), and rows and columns have both been sorted from most to least competitive.

	Q4	P9	P4	P8	P1	Q10	Q7	P5	Q5	Q3	P6	Q8	Q9	P7
**Q4**		4.15	5.49	14.08	19.61	27.78	28.57	27.03	37.04	20.83	40	29.41	62.5	21.74
**P9**	-4.15		0	9.01	13.16	15.38	22.22	16.95	18.18	14.08	23.81	23.26	26.32	16.67
**P4**	-5.49	0		4.22	5.21	12.2	14.93	15.38	16.95	10.75	27.78	21.74	29.41	14.49
**P8**	-14.08	-9.01	-4.22		0	1.63	12.99	11.36	6.21	7.58	20.41	9.35	25.64	11.24
**P1**	-19.61	-13.16	-5.21	0		2.74	8.62	6.1	6.94	3.02	16.67	8.55	21.28	18.18
**Q10**	-27.78	-15.38	-12.2	-1.63	-2.74		5.99	1.72	4.78	0	9.62	8	20	11.63
**Q7**	-28.57	-22.22	-14.93	-12.99	-8.62	-5.99		0	0	0	8.93	4.02	13.33	10.87
**P5**	-27.03	-16.95	-15.38	-11.36	-6.1	-1.72	0		0	0	2.83	4.05	8.7	10.87
**Q5**	-37.04	-18.18	-16.95	-6.21	-6.94	-4.78	0	0		0	6.06	1.93	16.95	7.63
**Q3**	-20.83	-14.08	-10.75	-7.58	-3.02	0	0	0	0		0	4.24	1.52	0
**P6**	-40	-23.81	-27.78	-20.41	-16.67	-9.62	-8.93	-2.83	-6.06	0		0	4.55	10.42
**Q8**	-29.41	-23.26	-21.74	-9.35	-8.55	-8	-4.02	-4.05	-1.93	-4.24	0		0	1.39
**Q9**	-62.5	-26.32	-29.41	-25.64	-21.28	-20	-13.33	-8.7	-16.95	-1.52	-4.55	0		0
**P7**	-21.74	-16.67	-14.49	-11.24	-18.18	-11.63	-10.87	-10.87	-7.63	0	-10.42	-1.39	0	

Figure R: Competition experiments between the 14 adapted populations of Q1–10 and P1–10 with leakage (cf. Fig Q). Two populations are inoculated side-by-side (one at the top, one at the bottom) of an enlarged grid which fits both populations entirely (50×275). Each competition experiment lasts for 10^5 timesteps and includes mutations. The time at which one population has expelled the other from the grid is recorded and a draw is recorded if both populations are still present on the grid at the end. All values in the table denote competitive success of the population in the row relative to the population in the column ($s = \frac{10^5}{t_{\text{win}}}$), and rows and columns have both been sorted from most to least competitive.

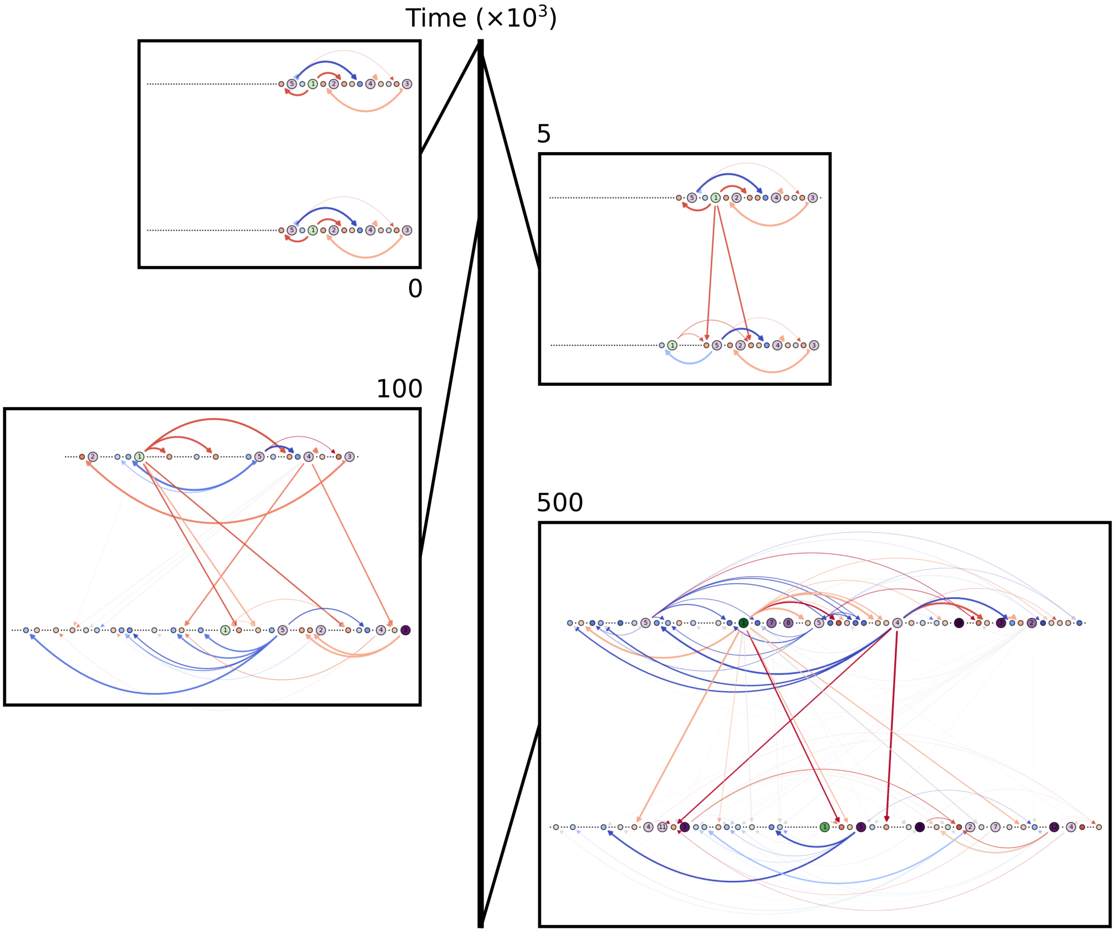

Figure S: Evolution of host control along the ancestral lineage of Q4.

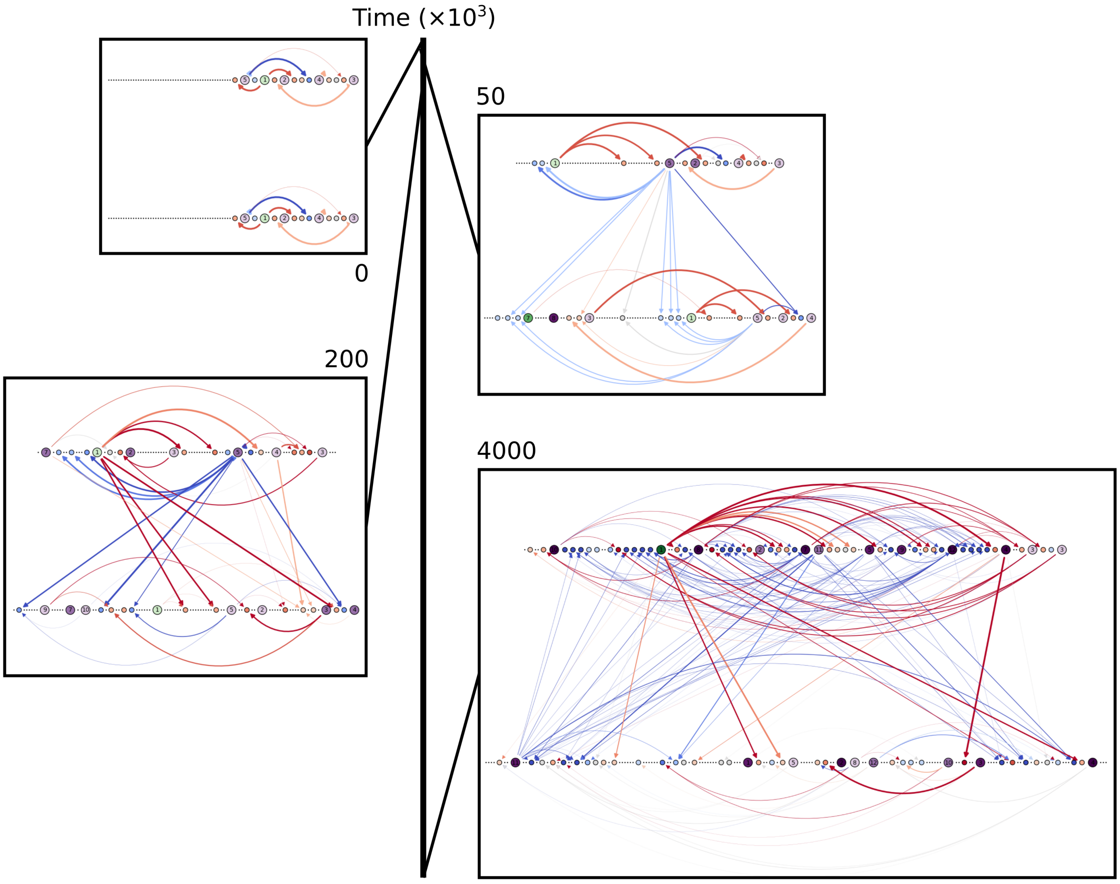

Figure T: Evolution of bi-directional control along the ancestral lineage of Q3.

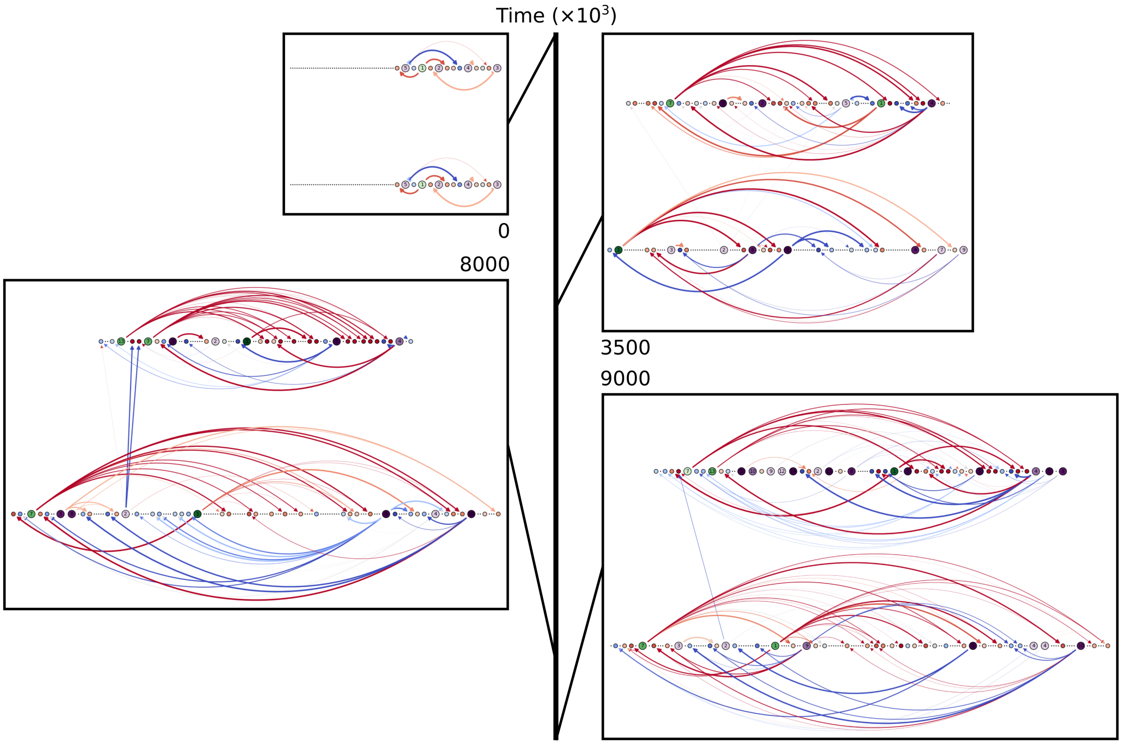

Figure U: Evolution of symbiont control along the ancestral lineage of $Q_{HS}10$.
