# Supplementary material for: Intracellular signaling in proto-eukaryotes evolves to alleviate regulatory conflicts of endosymbiosis
Source: PLoS Comput Biol. 2024 Feb 9;20(2):e1011860. doi: 10.1371/journal.pcbi.1011860 (PMC10883579; doi:10.1371/journal.pcbi.1011860)
Supplement: Attachment — Submitted filename: ResponseToPLoS.pdf [file pcbi.1011860.s002.pdf]

Response to the reviewers

We thank the reviewers for their detailed and constructive feedback. They appreciate the unique novelty and likely interest of our study, with Reviewer #2 for example writing: "Thus, the paper will interest to a wide audience of researchers interested in cell and evolutionary biology."
At the same time, a number of difficulties were raised. A few of these were mentioned by multiple reviewers: (1) the methods should be extended, (2) the analogy with cell size control is unclear, (3) some results are lacking references to data. These points have been addressed in the new version of our manuscript.

In addition, we would like to make the general remark that the aim of our study is to *explore* evolutionary behaviors of an endosymbiotic system, which is a crucial puzzle piece in understanding the origin of eukaryotes. There is little consensus on how the mitochondrial endosymbiosis precisely proceeded, and we do not wish to claim that our model can somehow accurately recapitulate what happened during eukaryogenesis. Yet the various behaviors that evolve in our simulations potentially capture different aspects of evolution that could play some kind of role in an obligate endosymbiosis: implicit control represents coordination through metabolism; explicit control coordination through regulation; synchronization links symbiont dynamics to the host cell-cycle which is found in many endosymbioses in nature; evolution of large host and small symbiont genomes also resembles endosymbiosis in nature. This is why in our manuscript we focus on mechanisms and great detail of a few evolutionary replicates rather than performing large-scale analysis of many evolutionary replicates (which would still not capture eukaryogenesis exactly).

All other points are addressed in our responses below. We reply with "check" when we comply with the minor changes suggested by a reviewer.

Reviewer #1: Understanding the coevolution of host and endosymbionts is interesting and the modeling approach taken here is potentially powerful. However, there are a few things that greatly temper my enthusiasm for the paper and having left me wanting to like this paper more than I do.

Why only 10 runs? I can't recall simulation-based analyses that rely on so few, and having only 10 and limits the ability to make generalizations. The few results means that broad generalizations are being based on (sometimes) only one or two outcomes. Does one (or two, or three) outcome out of 10 substantiate any generalization?
I assume the number of runs is so few because they are computationally demanding, but I have to assume, because the authors do not provide information on computational time (such information should be provided). Even if the simulations, as run, are computationally demanding I'm left wondering whether attempts were made to simplify / alter the parameters to run things faster. For example, it is common in population genetic simulations to increase the mutation rate and or strength of selection to explore some situations.

We would like to point out that the manuscript is in fact based on 50 replicates starting with *de novo* hosts and symbionts (see Fig. 3) and 144 replicates starting with pre-evolved hosts and symbionts (see Fig. S1). Relative to the single trajectory of eukaryogenesis that took place in nature, these replicates provide a wealth of data. Simulations are indeed demanding, with some of the main evolution experiments taking over 6 months to complete time trajectories of 10M. There is no obvious shortcut to this, because our model is not a

population genetic model. There is no explicit fitness criterion and ecology and evolution are in constant interplay; increasing the mutation rate leads to extinction. In the revised manuscript we have put more focus on the fact that alternative outcomes evolve in our model; and we explain that there is no explicit fitness criterion (last sentence of the first paragraph of "Holobionts adapt despite molecular interference").

The Results section is frustrating at times. Stories are told based on results of individual replicates, then broad claims are made about the consequences/effects of certain phenomena. But these claims are often made without clear reference to support those claims (and with so few replicates I don't know how the authors can be so certain). In other words, individual cases are being used to make broad claims (e.g. line 235), and the data supporting the claims are not always shown. Too many "For example….." (e.g. line 212),

As mentioned in our introduction to this reponse letter, we have taken care to provide our claims in the Results with data or references whenever these were previously missing. We have also clarified at several points in the manuscript that the description is specific to a particular replicate. Most broader claims (for instance how bi-directional control works) were based on inspection, experimentation and comparison of other replicates which were not explicitly discussed in the text; we have now added these data (e.g. as Supplementary Figures) or kept our discussion specific to one replicate only.

The Results discuss replicate Q4 fairly extensively and the authors point out that this is a highly successful symbiont. It is interesting if this is correct, although again, with only 10 replicates one doesn't know if Q4 really represents the best strategy. Moreover, I'm left wondering if the Q4 strategy is so successful, why is it seen in only one replicate? Are others trapped on local optima? Is mutation limitation preventing the other replicates from achieving a similar outcome? I would expect answers to such questions from a simulation study ….. even if not definitive answers, I would expect the authors to provide some insight.

We indeed state that Q4 is the most successful replicate out of all our replicates, but we point out that the three other control mechanisms also give rise to at least one successful replicate each. While we think Q4 is interesting in that it reflects certain aspects of endosymbiosis in nature, we have now amended our writing to also reflect the fact that there are various alternative trajectories, which all have the potential to achieve fit holobionts, and which all reveal different aspects of evolution in endosymbiosis.

We think that the diversity of evolutionary trajectories shows that our model contains various degrees of freedom (in particular no explicit fitness criterion), which, as in nature, we expect to allow for alternative solutions. Although complete rewiring of the gene regulatory network might be advantageous in some cases (i.e. to enhance efficiency of cell-cycle regulation), this becomes very hard once cells have become adapted with a specific regulatory network.

The authors seem focused on explaining the results of their simulations, but any ties back to what we see in the natural world are weak – even though the intro claims the simulations are motivated as a way to explore the early stages of eukaryotes. One example, is the paragraph starting on line 202, which starts with the sentence that "Host control creates selection for equal genome sizes…" and concludes with the sentence "Thus, cell-cycle synchronization promotes symmetry in genome size despite functional differentiation as observed before." But this is not the pattern we see in eukaryotes? So there appears a

mismatch between the lessons from the simulations and biological reality? If I'm correct, then what are we learning from this model?

Indeed, an important reason for the development of our model is to gain novel hypotheses on mechanisms, forces and outcomes at play in early stages of eukaryogenesis. We agree that no evolutionary replicate recapitulates eukaryogenesis, but we argue that our work uncovers multiple previously unknown aspects of obligate endosymbiosis, which may have been important at the origin of eukaryotes. As mentioned before, especially in the case of eukaryogenesis, which only took place once in nature, it is important to explore alternative trajectories. In eukaryogenesis, the host mostly took regulatory control over its symbionts (fitting with host control, though perhaps also with bi-directional control as there are still retrograde signals traveling from mitochondria to the nucleus), and the nuclear genome became much larger than the mitochondrial genome. We have found both these trends in our experiment, yet not in the same replicate, highlighting different reasons for their evolution and potential alternative outcomes of eukaryogenesis. As mentioned in the manuscript, precisely because genome size asymmetry does not emerge in the host control outcome in our model, it is worthwhile future research to explore several other features of obligate endosymbiosis which we did not include in our model, such as asymmetric transfer rates, asymmetry in mutation rates, etc.

Line 320. I find it puzzling that if the motivation is to understand the early stages of eukariogenesis the model is starting with identical host and symbiont genomes. The authors themselves acknowledge that the real world scenario would be better captured by having t=0 genomes of hosts and symbionts that are not identical? This adds to the general feel of the paper – that it is more interested in the results of the model than using the model to understand biology.

The reviewer is correct that the model that we focus on in our main text possesses several features that are very different from the obligate endosymbiosis at the heart of eukaryogenesis. Yet, starting with primitive and identical initial genomes allows us to model obligate endosymbiosis in a biologically interesting way, where host and symbiont are not yet entrenched in specific regulatory topologies and where leakage has a large effect on regulatory dynamics. In reality, the eukaryotic ancestors were of course not identical nor were they primitive, yet in our model formalism as we show, it turns out this is the best way to represent them to obtain the aforementioned behaviors (i.e. that the endosymbiotic conditions brings about cellular/informational challenges; shown in Fig. 5 and Fig. S1). As a nice side point, starting with identical host and symbiont means that we do not build in or impose too many other asymmetries in our model at the start (apart from the host—symbiont hierarchy and numeric asymmetry): any other asymmetry that appears, such as that in genome size or regulatory control, is emergent from the endosymbiotic condition itself.

Less substantive & minor issues:

Why are simulation data only "available upon request"? Most journals have moved away from this (and it is usually not long before it becomes quite difficult to obtain such data). Are the files too large to put on a publicly available repository?

We will make all data of our final populations for the 50 *de novo* replicates available online; we had initially decided against doing this because it is a lot of data and it is highly specific

to our model. Since the model code is also available online, people should be able to run (our) simulations from this data, and find everything that is represented in our figures.

Line 32 “relative to virtually all known.....which had already adapted to a endosymbionts (i.e. mitochondria)”. If there are endosymbiosis that are “known” that don’t involve a host with a mitochondria, then what are they (I’d like to know, and I assume other readers would too)? If non are known, then the “virtually all known” is not sensical.

We removed the word virtually.

Line 40 “..with one notable exception …”, good that there is a reference for this exception, but tell us what the exception is – if it is notable, then tell us, don’t make us go look at another paper.

Thanks, we have specified what this exception is.

Figure 1 provides a lot of data on a single replicate, and two other replicates are shown in the supplement. By why only 3? Why not provide these summary figures for all 10 replicates?

The most important panel in Figure 1 for our narrative is the bottom panel where we show how signaling emerges. In the Supplementary Material, we provide the same panel for all 50 *de novo* evolutionary replicates. We now also added complete summary figures for the other successful runs from Q1–10.

Figure 1 – the star apparently marks “the final population”….but it is not the final time in the simulation?

We have clarified this in the caption; the star marks the last common ancestor of the final population.

Line 125, I’m not at all convinced that 10 replicates of a simulation allows one to “evaluate the generality”

10 replicates is certainly not enough to quantify the probability to end up with some particular outcome (i.e. host control) or to ascertain that it is always the best. However, already our results show that host control is not the only possible outcome; there are three additional mechanisms for stabilizing endosymbiosis. Compared to the single trajectory of eukaryogenesis in nature, these outcomes already add new insight into our thinking about eukaryogenesis (even if not providing quantitative predictions).

Line 130 “the invention” of signaling? Perhaps origin? or evolution?

Check.

Line 165 “…the single outcome of eukariogenesis that appeared 1–2 billion years ago….” I thought the first eukaryotes were much older, and then estimate was much more precise than the billion year range. A reference would help.

We amended this statement and included the reference.

The paper would benefit from more references. For example, line 233 a claim is made about bi-direction control resembling a sizer mechanism for cell size control. But I don't know how sizer mechanisms work – a reference to a paper that explores the evolution of sizer mechanisms belongs here.

We noticed that our analogy with cell size control was unclear to multiple reviewers, and we think it distracts too much from our main story. Thus, we have decided to only discuss cell size control briefly in the discussion and in the supplement. In addition we have added several references to support this discussion.

Line 314 … "this is exploited by evolution…" but what is the selective advantage of transferring household [housekeeping] genes from symbiont to host?

The fact that the host executes a slower cell cycle which allows more time for replication of genes. In contrast, the symbiont cell cycle is slowed down by the necessity to replicate household genes. If these household genes are transferred from the symbiont to the host, then the symbiont can speed up its cell cycle, while the host can use its slow cell cycle to replicate more household geens. We clarified this in the manuscript.

Figures, horizontal axis .. .what is AUT?

Arbitrary units of time or timesteps, i.e. the unit of time of the simulation in which all cells are updated once; we clarified this at the first instance (Fig. 1).

Figure 4 caption….are these results from simulations with more distant genomes at t=0? Data are shown for 7 replicates, but how do I know which replicate belongs to which data?

No, these are *de novo* replicates evolved without leakage or transfer (the 7 surviving replicates out of P1–10, i.e. bottom row in Fig. 3). To allow the reader to link the replicates between Fig. 3 and Fig. 5, we have annotated the lines in Fig. 5.

Line 381. The authors report that the results presented here contrast with their "previous study (2023)"…but my understanding is that paper used the same model that was used here? So why the differing results? Seems odd the authors don't even try to explain this (if it is the same model, then perhaps having only 10 replicates?).

Thanks for pointing this out, we have now sharpened the text (also in the Methods indicating that our current model is an extension of the previous model). Our current model represents a different setting for eukaryogenesis where different evolutionary solutions arise for different evolutionary challenges; consequently, the impact on genome size asymmetry is also different, and the trend found in our previous work is not found in this work.

Line 425 "we use as a basis our previous model of obligate endosymbiosis and cell-cycle regulation (von der Dunk et al. (2023)," "as a basis" implies this is not the same model. If not, then how does this model differ? If the same model, then why "as a basis"

It is the same model framework but we have extended the model with leakage, transfer and targeting; we have sharpened our phrasing.

line 428. The model has 4 states, but only 2 are identified?

We have amended the sentence.

Page 2 supplement claims that “ implicit control is by far the most common evolved strategy in our model” BUT, what does this mean with regard to the biological relevance of this model?

Indeed the fact that implicit control appears to be a quite common attractor for obligate endosymbiosis in our modeling formalism is interesting. As mentioned, our goal is to explore the dynamics of obligate endosymbiosis in an evolutionary context, and for this finding alternative outcomes is highly informative. If the field were to one day reach consensus on the conditions and precise setting of the mitochondrial endosymbiosis, then we could try to model an exact replica of this evolutionary trajectory (of course, this requires many mechanistic details which even for extant organisms are not known) and by comparing with alternatives we could establish how unique the outcome of eukaryogenesis in nature really was.

At this point, matching biological reality should not be the main goal. These systems are so complex that one cannot expect to completely solve them with a single model. Moreover, modeling work on eukaryogenesis and even on endosymbiosis (which is more common in nature when we also include eukaryotic hosts) is scarce, and so we have here attempted to get some first insights into how cells could deal with informational challenges of obligate endosymbiosis. The biological relevance thus lies in the insights we have gained into this complex system: that coordination between host and symbiont is possible at various levels and with various specific regulatory mechanisms; that these mechanisms impact genome size evolution; and that various molecular challenges may influence the mechanism that evolves.

Page 3 supplement “strings of gene products that target more binding sites are more conserved than those of gene products with no or few regulatory targets (see “Gene family analysis” in Appendix 1 of Von der Dunk et al., 2022).” This is consistent with pleiotropic constraints, something seen with many signaling / gene / protein networks, the lack of reference to this literature is a bit concerning.

Thanks for pointing this out, we have added a reference.

Page 3 supplement “impact of leakage and transfer is largest when evolution is started with identical host and symbiont genomes, i.e. similar to the situation in the experiments described in the main text. When host and symbiont genomes start out identical, genes likely retain some of their ancestral overlap, resulting in the strongest molecular interference.” This is interesting, but what data support this claim?

Having read this comment, we tested our expectation, and found that indeed for gene g5, which is most constrained in sequence evolution due to usually having multiple binding site targets, the host and symbiont copy diverge very little in replicates starting with identical host and symbiont, whereas they are quite distinct in replicates starting with different host

and symbiont. We have added the data for this directly to the text in the Supplementary Results.

In general, the supplement has some interesting discussion, but the nearly complete lack of references (only two papers by these authors) and general paucity of data leave me now knowing if this is just overinterpretation of a few replicates of a single simulation model, or the claims are actually well supported.

We have added multiple references to figures and to literature in the Supplementary Results.

Figure S5, please repeat the meaning of the diamonds, triangles, colors, etc.

Check.

Reviewer #2: This paper by von der Dunk uses a computational model to explore mechanisms by which the coordination of cell cycle control between a host and endosymbiont can emerge during evolution. The study builds on previous work by the team, in particular on a previous paper by the same author on the evolution of cell cycle control.

The work is highly original and clever. There is no-one else in the world attempting this kind of analysis. The findings are interesting and promise to shed new light on the mechanisms and evolution of cell cycle control in the context of endosymbionts.

Thus, the paper will interest to a wide audience of researchers interested in cell and evolutionary biology.

The approach is interesting and sophisticated. The authors use a multilevel approach. Selection acts on the ability of holobionts possessing stochastic regulatory gene networks controlling DNA replication and division timing to populate a landscape with different levels of nutrients. During the course of evolution, the model allows for changes to genome architecture, gene transfer, changes to genes, their localization, and enhancers/promoters in both host and symbiont genomes.

The paper also attempts to dissect the effects of changes in i) the localization of regulatory machinery within the holobiont, ii) product leakage between host and symbiont, iii) and gene transfer between host and symbiont on the evolution, of successful holobiont propagation.

This leads to the interesting finding that while leakage creates problems via interference, it is a pre-requisite for the evolution of coordination - perhaps because genomes learn to live with one another.

After the evolution of successful holobionts, successful individuals are studied alone, in competition, and the role of leakage was assessed during their evolution.
All in all, the scope of the work is impressive!

In summary I would love to see a revised paper published.
However, in my view much needs to be done for it to make sense to an uninitiated reader.

We thank the reviewer for their positive summary of our work.

General comments:

1, The rules of the game

While one could imagine the model having been formulated in many different ways, the approach has been carefully thought through. The rules are sensible.
The title and abstract are appropriate.

However, the paper would benefit from more effort to explain the way the computational model is formulated. As it stands the paper is thin on details and requires readers to do a lot of work and sit with the previous paper Von der Dunk 2022 open while they read this paper.

In my view this is not good enough, the paper should stand on its own.
This is important because the assumptions of the model and the evolution affect the way one should think about the results.

We acknowledge this point, and now explain our methods more clearly and in more detail.

Details should be explained in a simple way, ideally in a table, and discussed in the paper. While I may not have fully gleaned all the assumptions (because they have not been made very clear), as I understand it, the following are some of the critical assumptions for the way the model works:

A. Mechanisms of cell cycle control:
The time taken for DNA replication to be completed depends on nutrient availability and genome size.
Thus, there is selection for holobionts that can delay division in a way that depends on nutrient conditions to avoid dividing with under-replicated DNA.
There is no way for organisms to monitor whether DNA replication is underway.
The only way they can measure DNA replication is by assessing whether specific genes in the genome (e.g. genes far from the origin of replication) are present in two copies.
They can also survive if they possess a cell division cycle that is longer than the time required for DNA replication.

All correct so far.

Question: Am I correct in thinking that once DNA is replicated it is assumed that cells never undergo a second round of DNA replication?

Yes this is correct.

Question: Is there is a corresponding change in dosage related to numbers of TF binding sites (i.e. competition for TF binding)?

Yes, the enitre genome is being replicated bead-for-bead, which means that entire loci including their binding regions can be found in two copies after replication.

B. Symbiosis:
Holobionts cannot live without symbionts. Symbionts use nutrients. This creates selection for reduced symbiont copy number.
Symbiont division is assumed to occur at the same time as the host. There is no way to evolve machinery to ensure their fair segregation (like that present in eukaryotes), symbionts are segregated randomly at division. As a result there is selection for increased symbiont number.

We think that the reviewer here means “Symbiont distribution” rather than “division” (which is in principle regulated independently by its cell cycle), in which case they are correct.

All symbionts are assumed to have the same genome.

This is not true, each symbiont is an individual in the model, which carries its own genome and mutations and executes its own cell cycle. We can see how this interpretation can

unfortunately arise from Figure 1, so we have adapted Figure 1 to show different symbionts.

C. Gene expression:
Gene expression is based on gene copy number. As a result, it also depends on symbiont copy number.
There is only one regulatory binding TF binding site upstream of each gene. This can only be functional or dead, there is no in-between state.

This is not true; a gene can have any number of binding sites upstream. Each of these are represented by a bitstring that defines with what affinity expressed genes are going to bind to them. Binding sites are discrete units treated as "beads-on-a-string" (clarified in the Methods) that can duplicate, delete and substitute. So binding sites and genes do not emerge *de novo* out of a piece of DNA, but their sequences are also not automatically functional (many binding sites and genes are not, or are only binding with low affinity). In fact, we chose our parameters such that two random sequences (random TF and random binding site) have a very low binding affinity, reflecting a non-functional binding site. Through point mutations, binding sites can change such that the binding affinity increases; thus, effectively binding sites can be anywhere in between junk (no gene has affinity for it) and functional (one or more genes has affinity for it).

Proteins can be targeted to symbiont or host or both based upon the signal sequence they possess.
Proteins can leak between symbiont and host. This leakage represents a fixed proportion of total protein.

Question: if TFs are targeted to both host and symbiont does this reduce their expression level?

There are no product concentrations, as our model is based on a Boolean regulatory network (Quinones-Valles et al., 2014); once expressed, a gene product is omnipresent in the cell. Dual targetted products are expressed in both compartments. These points have been made more explicit in the Methods.

Question:
What does this mean in the 2022 paper: "Noise is introduced through sequence-specific binding affinities between gene products and binding sites (where affinity is a function of bitstring similarity);"

See answer to "C. Gene Expression" above: the binding affinity is determined by the bitstring similarity between gene and binding site; only one gene binds a binding site (or no gene), and this is where noise comes in. A gene with high affinity for a binding site may still fail to bind every now and then, yielding stochasticity.

And I right in thinking that although expression is "stochastic", genes that are expressed are expressed in precise numbers that depend on genome copies, and when present in sufficient numbers act on all target genes?

Each gene can either be expressed or not; when expressed they can already target all binding sites, i.e. they join in the competition at each binding site. What is stochastic is

these binding interactions: depending on which genes bind to binding sites, the total excitation might be sufficient to activate a gene, leading to its expression. This means that given the same expression state, the binding interactions might not be exactly the same, and so the next state is not fixed.

Furthermore, the reviewer is right that extra genome copies lead to extra products. Thus, while there are no concentrations inside the cell, each gene copy that is expressed counts as an individual unit. Thus, a gene that has been replicated, may be activated and expressed at two loci, which increases its probability to bind at any binding site.

How much protein is required to have an effect on downstream targets following leakage?

Following the above explanation, leakage is also a stochastic process. Any expressed product may leak to another compartment with a small probability. If leaked, then the particular product becomes expressed in the other compartment as well (i.e. as if it were produced locally from that genome), and has the potential to bind all binding sites in that compartment. This was the most natural extension of our modeling formalism to incorporate leakage and transport. We hope that these details are now clarified in our methods.

D. Selection:

Please explain how population size affects selection in the model.

We would first like to point out that fitness and hence selection are not explicitly defined in our model (we have added this point to the first paragraph of the Results describing our experiments). Moreover, for spatial systems the relationship between population size and selection is not as well understood as it is for well-mixed models. We do note that in our *de novo* experiments, a few replicates go extinct because the population is small in the beginning and adaptation may not occur before a stochastic collapse of the population. With larger population size, extinction would be expected to be more rare. Furthermore, during this initial period we also note that genome sizes drift substantially because the population is so sparse that selection is likely very weak. Yet we did not further test for this link quantitatively.

Please explain how competition experiments differ from clonal experiments.

During competition experiments, two sets of holobionts are grown in the same grid, and we study which one outcompetes the other. In clonal experiments, a single type of holobiont is grown in a (small) grid to measure its carrying capacity, cell-cycle efficiency, etc. We have specified these experiments at their first appearance in the text.

Apologies if I should have understood this, but can the model select for organisms that grow slowly without making errors (persisters), or is the model formulated in such a way that those that grow fast always win out?

The model can in fact select for slow holobionts: the successful replicate P9 is a good example of this. Yet, because there is a small natural death rate, holobionts cannot become persisters completely. Moreover, dividing holobionts overgrow their neighbors which means that fast growers are expected to have an advantage over slow growers.

E. Solutions enabling survival:
Solutions include:
i). Both symbiont and host evolve a similar cell cycle that works independently in the two.
ii) The cell cycle time is longer than the DNA replication time in the host and symbiont.
iii) Symbiont genomes are small - since they only divide once per cell cycle this helps and doesnt lead to increased number. Increased numbers only arise from stochastic divisions.
iv) There is a checkpoint such that division cannot occur until gene dosage is high enough.

Are there any others?

We appreciate the reviewer's list of solutions, which contain several important insights of our model. A few remarks. Solution (ii) is general for all hosts and symbionts which manage to cope with their local nutrient conditions (if not, they die). Solutions (i) and (iii) seem to partly describe two aspects of the implicit control strategy, i.e. that host and symbiont evolve a cell cycle that works independently (although they in fact evolve quite different cell cycles) and that symbiont genomes are small and host genomes large (resulting from their different cell cycles and in line with the high abundance of symbionts). Solution (iii) also partly describes host control where symbionts only divide once per cycle (incited by the host) such that the only fluctuations in symbiont number derive from stochastic distribution at holobiont birth (i.e. synchronization). Yet, in the case of host control symbiont genomes are not smaller than host genomes, because their identical cell-cycle behavior results in pressure on equal genome size. It may be further pointed out that in all evolved control mechanisms, symbionts only divide once per cycle when the holobiont is in equilibrium, otherwise symbiont numbers increase or decrease with time. What differs between mechanisms is how holobionts return to the equilibrium when stochasticity at holobiont division results in more or fewer symbionts than the equilibrium; in the case of host control there is no intrinsic solution but holobiont-level selection simply weeds out individuals with too many or too few symbionts; in the other mechanisms coordination between host and symbiont stabilizes the endosymbiosis. Finally, solution (iv) is a general description of a cell-cycle checkpoint as we see it evolve in our model. Individually, host and symbiont may have evolved such checkpoint to respond to nutrient conditions (this happens for instance in the host control case in Q4; but see also our previous work on free-living “prokaryotes”: Von der Dunk et al., 2022). Holobionts with bi-directional control or symbiont control use a similar checkpoint to make the host sense the number of symbionts by being responsive to the copy number of a symbiont gene.

Other partial solutions / adaptations in our model that enable survival when facing leakage, are (v) becoming insensitive to product leakage, or, alternatively, exploiting product leakage in a cell-cycle checkpoint (either bi-directional control or symbiont control) to again measure the symbiont number, and (vi) evolving low symbiont number to prevent negative effects of leakage but also to maximise nutrient conditions and overall holobiont growth.

It is likely that this summary of the key assumptions and outcomes of the model contains errors.
Hence the need for clarity in the paper!

We thank the reviewer for their detailed feedback which really showcase the points in our Methods were unclear and we have improved the Methods extensively.

2. Figures and Figure legends

While there is much to praise in the paper and while the results are fascinating, the Figures and Figure legends are very confusing.

The first Figure should explain the approach - Figure 5.

Check (the model scheme is now Fig. 1).

Figure 2 should come before Figure 1.
Its weird to jump in with one that seems “eukaryotic like”

We chose and would prefer to start with a description of a single evolutionary replicate, in order to really guide our reader through the experiment. Moreover, as we discussed in the introduction to this response letter, our goal is to explore mechanistically the dynamics of obligate endosymbiosis in an evolutionary context (which we can glean from Fig. 2), not to give a quantitative overview (e.g. more like Fig. 3) of the possible outcomes of endosymbiosis. Thus, we first describe a single replicate, i.e. the most successful replicate, and then show the entire breadth of outcomes among 5 different evolution experiments, 50 replicates in total. In addition, we now also provide alternative trajectories of replicates Q1–10 and $Q_{HS}10$ in the Supplementary Figures.

For me, Figure 3 is incredibly hard to understand.

We understand that Figure 3 (now Figure 4) can be hard to understand, so we enhanced both visual clarity and extended the legend.

Figures should be clear and should be broken up into sections, e.g. a-d, so that the reader can follow what is where. They are too crowded.
They are more like slides in a powerpoint presentation than Figures in a paper.

Check.

Figure legends must explain what is in the Figure.
This is not the case. Much is left unexplained.
As it stands the Figure legends discuss conclusions.
This is the wrong place to do this.

We have amended our figure legends to describe more clearly what is being shown in each figure. Still, we think it is useful to state in the figure legend the main message being conveyed, linking to a specific part of our overall narrative.

3. Analogies.

A. The authors make assumptions about what eukaryotes like that appear at odds with eukaryotic cell biology.

In eukaryotic cells, mitochondrial DNA replication is uncoupled from host DNA replication. It occurs throughout the cycle. Mitochondria can also undergo multiple rounds of DNA replication each cycle.

In addition, mitochondrial scission is not coupled to coupled to mitosis.
Eukaryotic cells also possess mechanisms to ensure fair mitochondrial segregation.
Finally, mitochondria aren't a metabolic burden, they AID host cell growth.

These facts should be explained in the paper!

We fully agree with all these points and are sorry that perhaps because of the unclarity in the Methods section (see above), the reviewer is unaware that they are in line with our model.

In our model, replication of host and symbiont is also uncoupled, except in the case that regulatory signaling ensures that their cell cycles run in sync. However, in most control mechanisms, symbionts sometimes do multiple divisions before the host divides. If this continues for too long, the number of symbionts will increase, which in our simple model of unicellular eukaryotes is not beneficial (perhaps unlike specific tissues in multicellular eukaryotes). Furthermore, symbiont division is not coupled to holobiont division; it is the host (the nuclear genome) that regulates division of the holobiont, upon which all symbionts (which are in different stages of their own cell cycles) are randomly distributed to daugther holobionts. Although current eukaryotes possess mechanisms to ensure fair segregation of mitochondria, we do not want to assume such mechanism at the onset of the endosymbiosis. Interestingly, we conducted some experiments with the evolved holobionts (Fig. S2) where we tested their growth while enforcing symmetric distribution of symbionts, in line with the invention of a fair segregation mechanism (but we did not evolve such system). In all the four control strategies, symmetric divisions lead to a larger carrying capacity, suggesting that there would be selection for a fair segregation mechanism. Finally, while mitochondria are generally thought of as the energy factories of the cell, they also require resources. Eukaryotes do not carry an infinite number of mitochondria, so there is clearly some balance to be made. In our model, we chose for a very simple and agnostic way to define a balance: carrying no symbionts is fatal (the presence of a symbiont is required for the holobiont) whereas carrying more symbionts becomes increasingly burdensome because of nutrients.

B. The paper repeatedly compares results with cell size control models.
If the authors wish to do this, they need to explain the equivalence.
They also need to explain current thinking about cell size control, and this needs to reference the most recent papers on the subject.
Most eukaryotic and prokaryotic systems are currently thought to be adders.

As mentioned in a response to reviewer 1, we notice that multiple reviewers have remarks about our analogy with cell size control, which we think distracts from our main story. Thus, we have decided to only discuss cell size control briefly in the discussion and in the supplement.
While literature on cell size control indeed widely favors the adder mechanism, we do not find behavior analogous to that of adders in the case of symbiont number control. This is the reason that we do not put emphasis on adders and discuss timers and sizers which resemble mechanisms for symbiont control.

The text should properly explain models of cell growth and division.
1. Adder (add a fixed amount of mass per cycle).

2. Timer (divide after fixed time has elapsed). This never applies in systems that need to respond to environment. Only in development.
3. Sizer: (divide at specific size). Ensures that division is impacted by growth.

See above.

A key test of the cell size control mechanism used in cells is the study of the impact of both size on homeostasis, and the response time following size perturbations.
If the authors are serious about making the comparison with cell size, they should implement this by asking how the cell cycle is changed in cells with increased or decreased numbers of symbionts.

We think this would be a very interesting experiment. However, as stated above, we only wanted to point out cell size control mechanisms as an analogy / metaphore for the mechanisms of symbiont control in our model, and we believe that the analogy is currently distracting readers from our main points, so we have diverted it to the Discussion (and Supplement).

C. Line 225: There is a statement about cell ageing that is confusing.

As detailed in our response to the other comment to line 225 below, we have amended this part of the text.

D. Cell cycle checkpoints should be defined.

It is unclear to us whether the reviewer would like us to provide our definition of a cell-cycle checkpoint---which is no different from the common definition in cell biology literature---or that they would like us to define each cell-cycle checkpoint that we encounter (all of which emerge *de novo* and are thus not predefined)---which we do, and we in fact explain which genes are involved and which external information (e.g. symbiont numbers) is integrated by the regulatory network. In addition, we now mention cell-cycle checkpoints in the Methods and give brief background needed to understand how they work.

4. Statements not supported by data.

There are several cases in the paper where statements are made without the data being presented.
This is not appropriate. All instances of this should be fixed.

Check.

5. Signalling;

There was no experiment to allow leakage and transfer but no “signalling".
Why not? Please explain.

Our aim was to find out whether when targetting and transfer are possible some form of control (regulatory communication) would evolve. We did however also investigate the effect of having only leakage and showed it in the Supplement.

Specific comments on the paper:

Line 28:

It is not true to say that the nucleus has control over the mitochondria.
Mitochondrial DNA replication is not subject to host control, but is dependent on holobiont mass accumulation.

We acknowledge that strictly speaking there is no host control in extant eukaryotes, as there are also signals travelling from mitochondria to the nucleus (resembling more bi-directional control), and there is a lot of metabolic communication (more resembling implicit control). Because of these reasons, we now put less emphasis on host control as best representing eukaryogenesis.
That being said, it is abundantly stated in literature that mitochondria and their dynamics are linked in all sorts of ways to the eukaryotic cell cycle (e.g. see new references added to the manuscript: Capps et al., 2003; Scarpulla, 2011; Eisenberg-Bord and Schuldiner, 2017). Moreover, the replication machinery is encoded on the nuclear genome, and most biogenesis is likely performed by genes encoded on the nuclear genome (and thus under regulatory control thereof). Quantitatively, it still seems as though mitochondria are heavily regulated by the nucleus.

While scission occurs from the outside in many cases mitochondria, LECA likely had FtsZ:
https://www.pnas.org/doi/full/10.1073/pnas.1421392112

This is an interesting paper. Perhaps the reviewer feels that the host-derived mitochondrial division systems in model organisms mentioned in the paper resembles more closely our host control solution? Yet our model is agnostic about the origin of the division machinery (or for that matter, other effector proteins that are in reality active in each of the different cell-cycle stages). As long as the division machinery is encoded on and regulated by the nucleus (even if the components are the original bacterial components as in most non-model organisms as suggested by the paper), this is still in line with our host control scenario.

Line 43:
This is a hypothesis (without supporting references) and should be presented as such.

Check.

Line 59: This is not a good summary of cell size control models.
See above.

See our responses above.

Line 73: The text should;d describe the evolutionary experiment,
How was it done?
How many rounds?
How many individuals were analysed from each experiment?

We have extended the description of the evolution experiment at the beginning of the Results. Rather than rounds, we performed run the experiment for a specified number of

timesteps (10^7) and we analyse the detailed behavior of the most recent common ancestor of each replicate.

Line 74: Before diving into Q4, I'd like a summary of the results.

As mentioned in the introduction to this response letter, we prefer to first describe a single evolutionary replicate in detail, since those mechanistic details are what we are interested in most of all. To understand what population size and communication strategy means in Figure 3, we have to explain these for a single case, i.e. Q4.

Line 83: What do the authors mean by “selective forces are weak”?
Explain how population size and selection work in the model.

We mean that selective forces are weak at low population density and we have accommodated this in the sentence. As stated in our response to a similar remark above, there is no explicit description of the effect of population size and selection in our model.

Figure 1 Legend not clear enough. Check (now Fig. 2)
Needs to be divided up into sub Figures e.g. a-d. Check.
In the final part of the Figure, t=0 is insensitive to leakage – why?

The initial ancestors are not entirely insensitive to leakage, although much less sensitive than the ancestors at t=5000. This is due to the fact that the initial genomes are still identical and run the simplest shortest possible cell cycle (i.e. 4 timesteps total, one in each stage) such that leakage of products between host and symbiont only causes minimal disturbance.

Line 117: Where is data to support this statement about it being “essential” by t = 0.05?

By t=0.05, the signaling from host to symbiont has a positive effect on the population size of +0.19, which is the entire population size at that timepoint. This has been added to Fig. 2.

Line 118/119: What’s the evidence that leakage helps coordination?

We do not say that leakage helps coordination, just that there is communication through leakage in the beginning: however, this information transfer is of a bad kind, because it actually hurts the holobionts. Communication through leakage disappears as host and symbiont somehow evolve to be insensitive to leakage and, at the same time, evolve active communication (i.e. signaling) which helps their coordination.

Line 120-121: Please show evidence!

We added a reference to the later section where we show this.

Line 138 statement is too strong

We used strong phrasing because this is the main point of our paper, and because it follows so evidently from our results. Since we have already added the disclaimer “in our model” to

the sentence we do not see a good reason for weakening the statement that product leakage drives the evolution of host—symbiont signaling in our model.

Line 150-154: What does competition test? How do they compete? Is this different to growth speed?

We have added explanations of the clonal growth experiment and the competition experiments in the text. Competition tests the relative fitness of two entire populations (including mutants and mutational effects) across the entire nutrient gradient. Since hosts and symbionts interact with their environment (i.e. they have an affect on nutrient conditions), we do not know *a priori* what happens in direct competition between two populations where nutrient conditions may be slightly different from the ones experienced by each separately (even if we had measured their growth rate in their own conditions separately).

Figure 3: The Figure is incredibly hard to understand.

We have made several visual changes to Figure 3 (now Figure 4) in order to make it less overwhelming; the legend has also been extended.

The assumption is that host control is similar to eukaryotes.
This is not the case (see above). Eukaryotes have gene transfer. And coordination.
Moreover, the output of mitochondria affects cell growth and success.

As mentioned, we acknowledge this point, and now put more emphasis on the fact that we get alternative evolutionary outcomes all of which reflect different aspects of obligate endosymbiosis. As the reviewer mentions, mitochondria indeed possess direct feedbacks with the host cell, which may be considered at a similar level as implicit control. In real cells, there may be many more routes for coordination besides through regulatory communication. Nevertheless, it is generally accepted in literature that mitochondria are under cell-cycle control of the nucleus (in a way that of course depends on the species and the cell type in case of multicellular eukaryotes).

Line 197: Statement needs supporting data.
A good test of control would be to alter symbiont number and assay return to steady state.
This is how diagnostics of cell size models are tested.

We have added the data to the Supplementary Results. Fig. S2 shows that symbiont numbers are highly correlated in the case of host control. The higher death rate of holobionts in the case of host control (see text) then indicates that there is more holobiont-level selection. We can also note in Fig. S2a that when we enforce symmetric segregation of symbionts, the population quickly converges on having two symbionts per host, even though we started with the same symbiont distribution as seen on the left with stochastic segregation of symbionts (i.e. holobionts ranging between 2 and 18 symbionts).

Line 200: If its all selection then there is no control over symbiont number.
Is this the case?

There is control in the sense that the host in principle dictates the division of the symbiont at the same time of its own division (cell-cycle synchronization). This control is achieved at

the regulatory level. Yet, there is a lot of stochasticity, in particular in the segregation of symbionts at holobiont division---for which cells can not evolve a different mechanism---and there it is selection that ensures that holobionts end up with the equilibrium distribution of symbiont numbers again.

Line 205: This isnt how it works in eukaryotes.

True, this is not the case in eukaryogenesis, so the host control outcome of our model does not recapitulate eukaryogenesis completely.

Why shouldn't mitochondria replicate outside of DNA rep cycle of host, and have smaller genomes?

They just don't in the case of host control, which synchronizes the cell-cycle by integrating the regulatory networks of host and symbiont. Yet, in some of the other mechanisms symbionts do replicate at different times than the host, so we do not say that this is not possible. In our model, there is no incentive for symbionts to have smaller genomes than their host, unless it is to regulate a shorter cell cycle. In this case of host control however, host and symbiont take the same amount of time to replicate their genome, so there is no incentive to have a smaller symbiont genome. Again, as discussed before, many other factors might explain why mitochondria genomes are actually much smaller than the nuclear genome.

Line 220: Not clear how 2 fold change can be read out by stochastic system - please explain.

The formation of a cell-cycle checkpoint based on dosage changes has been explained briefly in the Methods now.

Line 225: This does not resemble ageing.
This resembles a normal cell cycle!!!

We agree that a normal cell cycle involves more than the expression changes that define the cell cycle in our model. We have edited the sentence. To contrast what happens in the case of bi-directional control with our strict definition of a cell cycle (the expression changes), we have now chosen the term life cycle to refer to the fact that developmental changes are going on inside the cell next to expression changes. That being said, it is not obvious to us why normal cells continuously increase their number of mitochondria or other intracellular compartments (are we talking about unicellular eukaryotes? In cell types of multicellular organisms, there is likely even more diversity…?)

Line 234:"usually" is overstatement.

We have rephrased the sentence.

Assumptions of the model should be presented clearly:

Line 243: Why is the timing of emergence of a network important? What does this tell us? All it means is that its hard to evolve, e.g. human intelligence.

We agree, this is the implication of what we observe here, i.e. that symbiont-to-host signaling evolves late (while host-to-symbiont signaling evolves very early).

Line 254: would be good to know how many of the solutions rely on quantitative gene expression before and after DNA replication.

The dosage changes caused by DNA replication are very important in all our evolved cells. In our earlier study of free-living "prokaryotes" (Von der Dunk, 2022), all 10 replicates evolved genome organization, although only some replicates were able to exploit the genome organization for a novel property, i.e. plasticity in the timing of the cell cycle. In Q4, the host cell cycle (which also controls the symbiont cell cycle) is also plastic, indicating that it formed a cell-cycle checkpoint based on the integration of genome organization with regulatory behavior. We already pointed out in the manuscript that bi-directional and symbiont control are also relying on the changes in gene dosage. Quantitative gene expression is important in all our solutions supporting our earlier finding that genome organization is a very important dimension for understanding gene regulatory networks.

Line 267: please show data supporting dormant state.
How was this studied?
What about other systems?

We have rephrased this because we meant that the state appeared dormant due to the absence of gene expression.

Line 320: I really like this experiment.

Thanks.

A modified Figure 5 should be included as Figure 1.

We have changed the order of our main sections in order to make our methods clearer from the outset.

Supplement:

What does the following mean?
"we setup an evolution experiment with 12 different host–symbiont pairs"
"Each host–symbiont pair was evolved in two or three separate replicates to untangle the effect of specific pairings."

It means that we started evolution experiments in triplicates of unique initial host—symbiont combinations. For every host—symbiont combination, three replicates were run which only differed in their random seed. Because there is a lot of variation between all runs, we can use the triplicates to get an idea of the general outcomes for each of the initial host—symbiont pairs. This has been clarified in the text.

Figure S1 - what is "Standard"?

This means without leakage and/or transfer, we have added this to Fig. S1.

In supplement it says:
"Thus, if we combine these results with those from the experiments described in the main text, implicit control is by far the most common evolved strategy in our model. Signaling only emerges when relatively primitive hosts and symbionts (i.e. which are not adapted to poor nutrient conditions) are exposed to product leakage from the start."

The Supplementary text should explain exactly what the results are and how experiments were done.This is discussion not supplement.

We already state in the main text that implicit control is the most common outcome across the set of 50 replicates discussed there. In the supplement, we discuss another set of replicates, where we also find implicit control. Since we do not want to put too much emphasis on the frequency of different outcomes of obligate endosymbiosis, we have removed this statement from the Supplementary Results. Note that in the main text, implicit control is already discussed as the most common evolved strategy so our narrative in the main text remains unchanged.

It says this:

"The inability to evolve signaling might be an important reason why holobiont popu-lations remain smaller in the presence of product leakage in particular"
Please explain.

This statement was indeed unclear, and has been edited. It is not specific for these replicates that the impact of leakage is lasting; most replicates evolved with leakage evolve smaller populations.

Reviewer #3: The manuscript by Hogeweg and co-workers seek to explain how cell-cycle synchronization evolved for a host-symbiont system. The system starts with an obligate mutualism (they cannot live without each other) but without any influence on each other's multiplication. Influencing the cell cycle of the other cell(s) can come about by implicitly leaking some peptides or targeting signals toward the other cell. The results show that multiple paths toward efficient cell-cycle control exists.
I like the manuscript; it is well written, and the results are explained in detail. My criticisms involve the presentation, both of which can be easily remedied.
(1) Do not use PLOS strange Intro-Results-Discussion-Method structure, I never understood why use it for theoretical papers which cannot be understood without going through the methods first. So structure the paper the usual way: Intro-Methods-Results-Discussion.

We happily oblige.

(2) My usual quibbles with manuscript heavily building on another paper is that the method section does not stands on its own, and the reader has to go to the other paper to understand what is going on. I think repetition in the method section should not be a problem between papers. For example, I was unable to find the initial number of genes in the host and the symbiont. What is the probability of deletion or duplication of a gene? What is the difference between the host and the symbiont (symbiont being smaller I presume)?
In summary, the manuscript should be accepted for publication with the above edits.

We also acknowledge this point, and have added much more detail to the method section. (There is no size difference between the host and symbiont, in our main evolution experiments, we start with identical symbiont and host genomes so they start out with the same number of genes, same cell cycle time, etc.).

Minor comments
Line95: there is a duplicate of "whereas"

Check.

L103. The subscript influx should not be italicized

Check.

L469 The subscript s should not be italicized

Check.

L474 this sentence reads bad, maybe the preposition "in" should be "of"

It specifically needs to be "consists in" here because we mean "interference exists in the form of gene transfer" rather than "interference consists of gene transfer, process X, and process Y" which is the proper use of "consists of".

L477 The subscript T should not be italicized

Check.

Have the authors made all data and (if applicable) computational code underlying the findings in their manuscript fully available?

We will make all relevant data for the 50 main evolutionary replicates of our manuscript available, even though the formats are highly specific for our model code. In addition, the model code is already available in github, which allows readers to run (our) simulations and perform the experiments used in our manuscript (i.e. clonal growth experiments, competition experiments).

The PLOS Data policy requires authors to make all data and code underlying the findings described in their manuscript fully available without restriction, with rare exception (please refer to the Data Availability Statement in the manuscript PDF file). The data and code should be provided as part of the manuscript or its supporting information, or deposited to a public repository. For example, in addition to summary statistics, the data points behind means, medians and variance measures should be available. If there are restrictions on publicly sharing data or code —e.g. participant privacy or use of data from a third party— those must be specified.

Reviewer #1: No: Results are "available upon request" ...not sure if that is OK.

Reviewer #2: No: Many statements arent supported by data

Reviewer #3: Yes

PLOS authors have the option to publish the peer review history of their article (what does this mean?). If published, this will include your full peer review and any attached files.

If you choose “no”, your identity will remain anonymous but your review may still be made public.

Do you want your identity to be public for this peer review? For information about this choice, including consent withdrawal, please see our Privacy Policy.

Reviewer #1: No

Reviewer #2: No

Reviewer #3: Yes: Ádám Kun
